# Carcinoma-Derived Interleukin-8 Disorients Dendritic Cell Migration Without Impairing T-Cell Stimulation

**DOI:** 10.1371/journal.pone.0017922

**Published:** 2011-03-14

**Authors:** Carlos Alfaro, Natalia Suárez, Ivan Martínez-Forero, Asís Palazón, Ana Rouzaut, Sarai Solano, Esperanza Feijoo, Alfonso Gúrpide, Elixabet Bolaños, Lorena Erro, Juan Dubrot, Sandra Hervás-Stubbs, Alvaro Gonzalez, Jose Luis Perez-Gracia, Ignacio Melero

**Affiliations:** 1 Gene Therapy and Hepatology Division, Centro de Investigación Médica Aplicada (CIMA), Pamplona, Spain; 2 Biochemistry Department, Clínica Universidad de Navarra, Pamplona, Spain; 3 Medical Oncology Department, Clínica Universidad de Navarra, Pamplona, Spain; University of Nebraska Medical Center, United States of America

## Abstract

**Background:**

Interleukin-8 (IL-8, CXCL8) is readily produced by human malignant cells. Dendritic cells (DC) both produce IL-8 and express the IL-8 functional receptors CXCR1 and CXCR2. Most human colon carcinomas produce IL-8. IL-8 importance in malignancies has been ascribed to angiogeneis promotion.

**Principal Findings:**

IL-8 effects on human monocyte-derived DC biology were explored upon DC exposure to recombinant IL-8 and with the help of an IL-8 neutralizing mAb. *In vivo* experiments were performed in immunodeficient mice xenografted with IL-8-producing human colon carcinomas and comparatively with cell lines that do not produce IL-8. Allogenic T lymphocyte stimulation by DC was explored under the influence of IL-8. DC and neutrophil chemotaxis were measured by transwell-migration assays. Sera from tumor-xenografted mice contained increasing concentrations of IL-8 as the tumors progress. IL-8 production by carcinoma cells can be modulated by low doses of cyclophosphamide at the transcription level. If human DC are injected into HT29 or CaCo2 xenografted tumors, DC are retained intratumorally in an IL-8-dependent fashion. However, IL-8 did not modify the ability of DC to stimulate T cells. Interestingly, pre-exposure of DC to IL-8 desensitizes such cells for IL-8-mediated *in vitro* or *in vivo* chemoattraction. Thereby DC become disoriented to subsequently follow IL-8 chemotactic gradients towards malignant or inflamed tissue.

**Conclusions:**

IL-8 as produced by carcinoma cells changes DC migration cues, without directly interfering with DC-mediated T-cell stimulation.

## Introduction

In a previous study [1] we showed that ^111^Indium-labeled DC when injected into tumor lesions of patients suffering advanced digestive carcinomas [2] tended to remain inside the injected lesion. An explanation for such a retention was proposed in the sense that the human tumors abundantly produce IL-8 [1], [3] and DC express CXCR1 and CXCR2 functional IL-8 receptors on their plasma membrane [1], [4], [5]. However, no definitive proof was provided for the role of IL-8 in intratumoral retention of DC [1].

An IL-8 homologue is absent from the mouse genome and these precludes incisive definitive genetic experimentation on the role of IL-8 in murine tumor models. However there are reports suggesting that mouse CXCR1 is activated by human IL-8, hence permitting to some extent experiments in xenografts [6].

Chemokine receptors guide DC in physiology and in inflammation [7], [8]. DC migration from inflamed/infected [9] or malignant tissues [10], [11] is important for the orchestration of immune responses. Chemokine receptors do not only regulate motility but also control other cellular functions such as activation or survival in various cell types [11], [12]. Therefore it would not be a surprise if the chemokine microenvironment modified DC functions other than migration [12].

Human tumor cells produce IL-8 in most cases [1], [13] as a biological dirty trick played by the malignant tissue to promote angiogenesis [3], [13], [14], [15] and possibly to support the type of smoldering inflammation that promotes tumor progression and metastasis [14], [16], [17]. Tumor growth in human patients statistically correlates with IL-8 serum concentrations [3], [18]. Recently, a role for IL-8 has been described in the resistance to antiangiogenic VEGF signal blockade with sunitinib [19]. Importantly escape from sunitinib can be thwarted by co-treatment with neutralizing anti-IL8 mAb [19].

IL-8 was originally discovered as a powerful attractor of polymorphonuclear leukocytes (PMNs) [20], [21] in acute inflammation [21], but may act on other leukocyte subtypes [1], [22] and on endothelial cells [15]. In turn, DC are both responsive to IL-8 [4], [5], and produce IL-8 either when inactive or more overtly so, when activated/matured [1]. Injecting DC inside tumors has been intended to enhance antitumor activity for therapeutic purposes in animal models [11], [23] and in the clinic [2], [24], [25]. One of the hurdles faced is that the tumor microenvironment is rich in substances impairing DC functions [11], [26]. DC migration into lymph nodes is of critical importance in cancer immunotherapy based on DC [27], [28], [29]. If retained intratumorally, DC would be prey for tumor microenvironmental factors such as TGF-β for longer periods of time [11], thereby causing damage to the induction of anti-tumor immunity.

Here we show that xenografts of human tumors retain DC inside the injected tumors by means of IL-8-mediated chemoattraction, that can also recruit DC to the malignancy when injected in the subcutaneous connective tissue in the vicinity of the tumor. However, the same functional recombinant IL-8 that attracts DC and PMNs does not impair the abilities of DC to induce T-cell activation and proliferation either *in vitro* or *in vivo*. Interestingly, pre-exposure of DC to IL-8 restrains subsequent migration towards IL-8 chemo-attractive gradients indicating desensitization of the receptors.

## Methods

### Ethics statement

Animal studies have been performed in accordance with Spanish legislation under specific approval from the institutional ethics board by the *Comité de Ética para la Experimentación Animal of the University of Navarra* (Study 03/007 approval). Human cells are obtained from Blood donors (public blood bank of Navarra) under written informed consent for research.

### Dendritic cell generation

Dendritic cells were generated from filter buffy coats (FBC)-derived monocytes donated by healthy donors [30] who explicitly sign a written informed consent. To generate immature DCs from monocytes, human peripheral blood was isolated by Ficoll-Paque gradient centrifugation from FBC. Isolated mononuclear cells from these sources were subjected to positive selection using anti-CD14-conjugated paramagnetic beads and purified using the AutoMacs system according to the manufacturer's instructions (Miltenyi Biotec, Bergisch Gladbach, Germany). Purified monocytes were cultured for 7 days in RPMI-1640 with 5% (v/v) heat inactivated FCS. To differentiate dendritic cells from CD14^+^ cells, culture medium was supplemented with GM-CSF (1000 U/mL; Novartis, Basel, Switzerland) and IL-4 (500 U/mL; R&D Systems, Minneapolis, MN). DCs were matured adding clinical grade TNF-α (50 ng/mL; Boehringer Ingelheim, Ingelheim, Germany), IFN-α (1,000 IU/mL; Schering-Plough, Kenilworth, NJ) and Poly I:C (10 µg/mL; Ampligen, Bioclones, Tokai, South Africa) for 48 h.

### Cell lines and IL-8 concentrations in mouse serum and culture supernatants

HT29, CaCo2 and SW48 colon carcinoma cell lines were obtained from American Type Culture Collection (Rockville, MD). Cell lines were cloned by limiting dilution in 96-well plates and subcultures (10^5^ cells) were tested for the concentration of IL-8 in the 24 h supernatants from these subcultures by means of ELISA (BD Biosciences, San Diego, CA). Cyclophosphamide (Cytoxan) was purchased in our hospital pharmacy.

### Semiquantitative RT-PCR for IL-8

Total cellular RNA was extracted with Trizol (Invitrogen, Carlsbad, CA) according to the protocol provided by the manufacturer. First-strand cDNA was synthesized from 1 µg total cellular RNA using an RNA PCR kit (Takara Bio Inc., Otsu, Japan) with random primers. Thereafter, cDNA was amplified using 30, and 28 cycles for IL-8 and for β-actin, respectively. The specific primers used were as follows: IL-8, forward primer 5′-ATGACTTCCAAGCTGGCCGTG -3′ and reverse primer 5′-TTATGAATTCTCAGCCCTCTTCAAAAACTTCTC-3′; and for β-actin, forward primer 5′-GTGGGGCGCCCCAGGCACCA-3′ and reverse primer 5′-CTCCTTAATGTCACGCACGATTTC-3′. The product sizes were 300 bp for IL-8, and 548 bp for β-actin. The thermocycling conditions for the targets were as follows: denaturing at 94°C for 30 s for IL-8, and β-actin, annealing at 60°C for 30 s for IL-8 and β-actin, and extension at 72°C for 90 s for IL-8 and β-actin. The PCR products were fractionated on 2% agarose gels and visualized by ethidium bromide staining. The quantity of a band was measured by the area under its intensity profile curve using BioRad Quantity One 1-D Analysis Software (Bio-Rad Laboratories, Hercules, CA, USA). β-actin was employed to normalize the amount of RNA used in each reaction.

### Mouse tumors

Nude mice, Rag^−/−^ or Rag^−/−^ IL-2Rγ^−/−^ were obtained from The Jackson Laboratory. Animal experiments were in accordance to Spanish laws and approval was obtained from the animal experimentation committee of the University of Navarra (Study 03/007 approval). These mice were injected with the tumor cell lines HT29 (5×10^6^ cells), CaCo2 (10^7^ cells) or SW48 (5×10^6^) to induce subcutaneous tumors. IL-8 in serum samples was sequentially measured by ELISA (BD Biosciences). When indicated 3 mg/mouse of cyclophosphamide were injected i.p.

### *In vivo* migration

Female nude mice, Rag^−/−^ or Rag^−/−^ IL-2Rγ^−/−^ as indicated, were subcutaneously injected with 5×10^6^ HT29 (n = 4), 10×10^6^ CaCo2 (n = 4) or 10^6^ SW48 (n = 3) tumor cells. When tumors reached about 1 cm diameter, 10^6^ mature DCs were labelled with 2.5 µM CFSE (Sigma, Barcelona, Spain) or 4×10^−6^ M PKH26 (Sigma), washed and injected intratumorally. Mouse IgG (100 µg, BD Pharmingen) or neutralizing anti-human IL-8 mAb (100 µg, BD Pharmingen) were coinjected within the same syringe into the tumors. In case of HT29 and SW48, cell suspensions from the tumors were generated with the GentleMacs dissociator device (Miltenyi Biotec). Cell suspensions were analysed by FACS and fluorescent cells counted. In the case of CaCo2 xenografts, three days later, tumors were mechanically homogenized. Tissue homogenates were cleared from debris by centrifugation and fluorescence was measured using a plate fluorimeter (Polarstar Galaxy, BMG). Migration was calculated as fluorescence in the tumor divided by total input fluorescence injected (fluorescence was quantified in arbitrary units).

### Cytokine production by maturing DC

For *in vitro* stimulations, 10^5^ DC were cultured 48 h with medium alone (control), LPS (1 µg/mL) purchased from Sigma, R-848 imidazoquinoline (1 mM) purchased from Pharmatech (Shangai, China) or sCD40L at 200 ng/mL purchased from Abnova (Taipei, Taiwan). After culture for 48 h, supernatants were collected and the cytokine concentration was determined by immunoassay. Commercially available ELISA kits were used for the detection of IL-12p70 and IL-10 (BD Bioscience).

### FACS analysis

FITC and PE-labeled mAb specific for the DC maturation markers: CD80, CD83, CD86 and HLA-DR (BD Bioscience) and isotype-matched labeled controls were used to characterize cell surface phenotypes by flow cytometry. Dendritic cells (10^5^) were washed in cold PBS and incubated 15 min at 4°C with specific FITC or PE-labeled Abs. MAbs against IL-8 receptors (CXCR1 and CXCR2) were used by indirect fluorescence developed with a rabbit anti-mouse antiserum tagged with FITC (Jackson ImmunoResearch Labs, West Grove, PA).

### *In vitro* and *in vivo* MLR

*In vitro* MLR were performed as described [26]. Briefly, a total of 2×10^5^ lymphocytes from a distinct donor were added on day 9 at different T cell∶DC ratios (1280∶1, 640∶1, 320∶1, 160∶1, 80∶1 and 40∶1). After 3 days, the [methyl-3H]thymidine uptake was determined by the addition of 1 µCi of [methyl-3H]thymidine.

Female Rag^−/−^ IL-2Rγ^−/−^ were subcutaneously injected with 5×10^6^ HT29 cells. When tumors reached approximately 1 cm in diameter, these mice and tumor-free mice were intraperitoneally injected with 1×10^6^ DC and 5×10^6^ PKH2-labeled PBLs. After 4 days, cells were obtained by intraperitoneal lavages and samples were analysed using a FACSCalibur Flow Cytometer (Becton Dickinson). The number of T cell divisions is proportional to the dilution of PKH2 intensity and was found to be negligible in the absence of DC (data not shown). For FACS analysis lymphocytes were gated based on FSC/SSC features.

### PMN purification and fluorescence labelling

*In vitro* neutrophil and DC migration was measured in Transwell Chambers (5 µm; Corning Costar, Corning, NY). PMN cells were enriched by sedimentation of peripheral blood mixed in a dextran (6% v/v) solution. After sedimentation, floating fractions were collected. Red cells in the resuspended pellets were osmotically lysed. The remaining cell suspensions were layered onto Ficoll-Paque gradients and pellets were collected and washed after centrifugation. Neutrophil purity was >95% (CD15^bright^ neutrophils)

### *In vitro* chemotaxis assay

*In vitro* neutrophil and DC migration was measured in Transwell Chambers (5 µm; Corning Costar, Corning, NY). Both PKH26-DCs (10^5^) and PKH2-labeled neutrophils (10^5^) or only PKH2-labeled neutrophils were added to the upper chamber and migration stimuli were placed in the lower chamber. In this experiment IL-8 (R&D Systems) was used at 20 ng/mL as positive control. In other cases PKH26-DCs with or without IL-8 neutralizing mAb or IgG as control (BD Pharmigen) at 20 µg/mL was placed in the lower chamber as indicated. Transmigrated cells in the lower chamber were quantified using a FACSCalibur flow cytometer (BD Biosciences) or fluorescence microscopy imaging of the lower chamber. In some cases the lower chamber contained a subconfluent monolayer of HT29 cells. The chemotactic index was calculated as the number of migrated cells in the experimental conditions divided by number of migrated cells in the negative control, which is complete culture medium. In the experiments with HT29 cell in the lower chamber number of PKH2-fluorescent DC per microscopic field (×20) in the lower chamber were quantitated in triplicate wells by a blinded observer. Recombinant MIP3α was from R&D.

### Statistics

Comparisons were made with paired student's t tests. Values of p are given in the corresponding experiments.

## Results

### HT29 and CaCo2 tumor cell lines xenografted into immunodeficient mice generate tumors that produce IL-8

**Figure 1:**
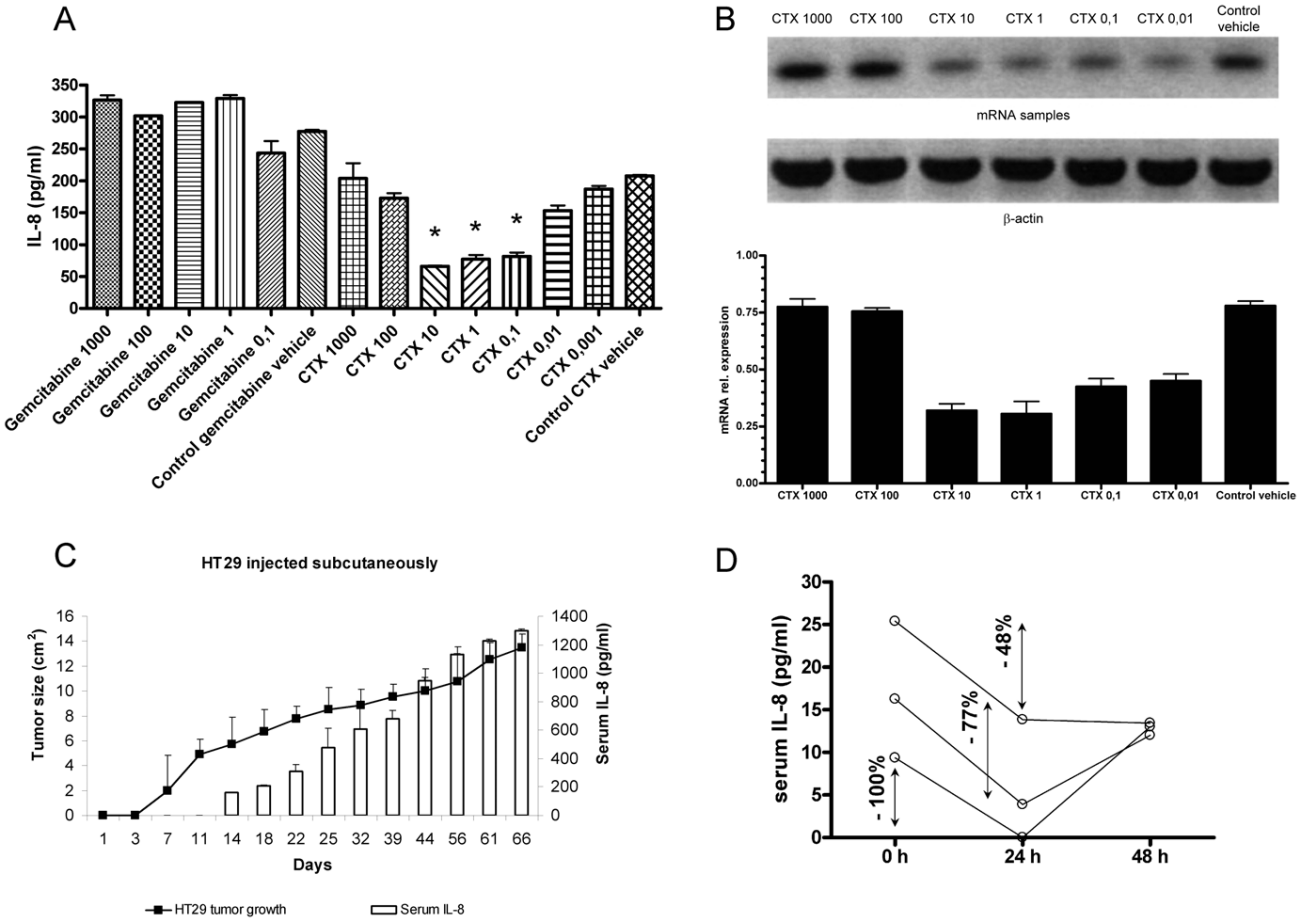
HT29 cells when xenografted secrete IL-8 to the plasma of the mice. ELISA determination of IL-8 in the supernatant of HT29 confluent cultures in the presence of indicated concentrations of cyclophosphamide (1000 to 0.001 µg/mL), or gemcitabine (1000 to 0.1 µg/mL) during the 24 h prior to supernatant collection. When indicated the solution vehicle of both drugs was added at the highest concentration. Results represent mean±SEM from three experiments. (B) Separate set of experiments as in A but in this case HT29 were collected and IL-8 mRNA was quantified by RT-PCR. PCR bands are shown in the upper panel and densitometry quantitative data given in the lower panel representing relative expression of IL-8 mRNA in comparison with β-actin mRNA. (C) Subcutaneously xenografted HT29 cells in Rag^−/−^ IL-2Rγ^−/−^ mice gave rise to progressing tumors (left axis depicting mean tumor diameter) and increasing serial serum concentrations of human IL-8 (right axis). Results represent mean±SEM of three independent experiments with 6 mice per experiment. Similar results were observed with CaCo2 tumors xenografted in the peritoneal cavity (Figure S1). (D) IL-8 serum concentrations of three individual Rag^−/−^ IL-2Rγ^−/−^ mice bearing HT29 tumors for seven days before a single intraperitoneal injection of 3 mg of cyclophosphamide and 24 and 48 h after treatment. The percentages of reduction at 24 h are indicated. Experiments were repeated at least three times with the exception of (D) that was performed with three individual animals.

A panel of human colon carcinomas was tested in order to identify cultures that produce high amounts of IL-8 to the supernatant [1]. All clonal subcultures of HT29 showed high homogeneous outputs of IL-8 while the SW48 cell line did not reach detectable levels in any experiment, and CaCo2 subcultures showed around one half of the production when compared to the levels attained by HT29 cells cultured at identical density for the same period of time (Figure S1). Microenvironment conditions and therapy may modify the ability of tumor cells to produce IL-8. Figure 1A shows that the production of IL-8 secreted to culture supernatants by viable HT29 cells was reduced in 24 h by exposure to low concentrations cyclophosphamide, while cells still preserved membrane integrity (>90% viability by trypan blue exclusion). Interestingly, the low range of cyclophosphamide concentrations was more effective at preventing IL-8 bioproduction and secretion to the supernatant. Gemcitabine and radiation did not or more weakly affected IL-8 secretion (Figure 1A and data not shown). Moreover, in a repeated set of experiments semiquantitative RT-PCR for IL-8 in comparison with the house keeping mRNA β-actin showed that cyclophosphamide inhibits IL-8 production at the mRNA level in a dose-dependent manner (Figure 1B), in such a way that low dose cyclophosphamide was better at mediating this effect than higher concentrations.

These results open the possibility that IL-8 production can be acutely reduced by cyclophosphamide for therapeutic purposes. Indeed, metronomic cyclophosphamide is becoming an attractive alternative for cancer management [31] and potentiation of a variety of immunotherapies [32].

When HT29 was xenografted in athymic nude mice, it gave rise to subcutaneous nodules that grew steadily over time (Figure 1C). Sequential sera samples from such animals contained increasing concentrations of IL-8 (Figure 1C) that correlated with tumor progression as reported in human patients [3].

CaCo2 failed to graft as subcutaneous nodules in two thirds of cases (data not shown), but grafted homogeneously as multiple peritoneal nodules if injected intraperitoneally (Figure S2). CaCo2-grafted animals also showed circulating IL-8 (Figure S2) but at lower concentrations if compared to HT29-bearing mice, as expected from the productions of IL-8 in the cell line cultures. Apart from this quantitative difference, the tendency was similar in tumors from both cell lines.

Importantly, treatment of mice with a single dose of 3 mg/mouse of cyclophosphamide reduced the serum concentration of IL-8 in the next 24 h in a range from 48 to 100% (Figure 1D), while those concentrations rapidly rebound in 48–72 h. It is of note that for this experiment mice with 7-day palpable tumor xenografts were used, so the concentrations of IL-8 in plasma were still low.

In conclusion, xenografted colon carcinomas retain the property of producing high amounts of human IL-8, and our results indicate that such a function could be modified by cyclophosphamide.

### Exogenously injected human DC inside xenografted HT29 tumors are retained by IL-8 in the tumor microenvironment

In order to study whether IL-8-producing tumors would retain intratumorally DC injected inside the lesion, we first chose the HT29 xenografts because of their higher bioproduction of IL-8.

**Figure 2:**
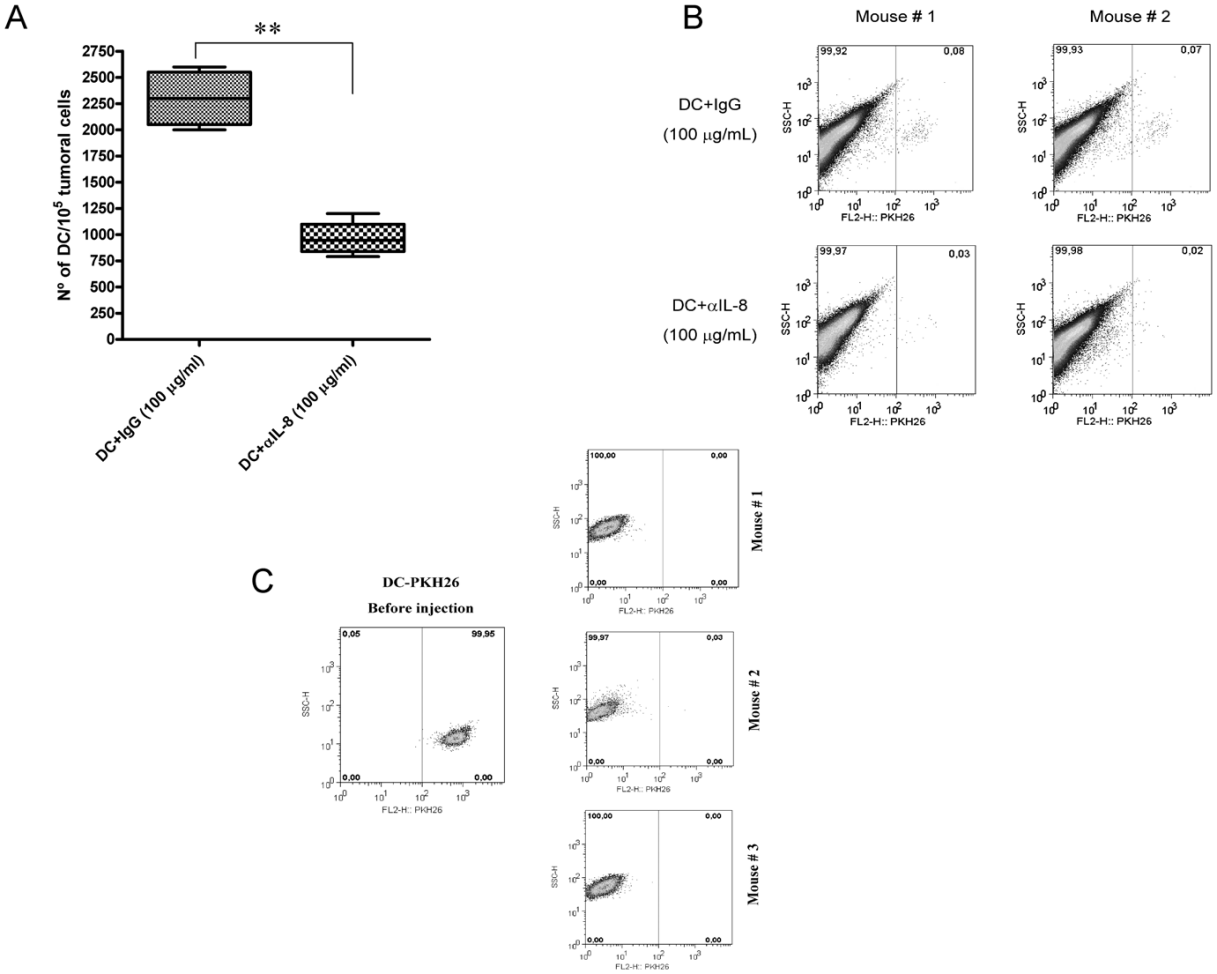
IL-8 produced by tumor cells *in vivo* retains DC inside xenografted tumor nodules. (A) HT29 was xenografted into Rag^−/−^ IL-2Rγ^−/−^ double KO mice. Tumor nodules, 8–12 mm in diameter, were injected with 5×10^6^ PKH26-labeled monocyte-derived DC. When indicated, the 100 µL DC suspensions contained 100 µg/mL of mouse IgG (control antibody) or anti-IL-8 neutralizing mAb. The figure shows the proportion of PKH26^+^ events with respect to total tumor cells upon FACS analysis, three days after DC injection. Four mice with two bilateral xenografted tumors each per condition were used. Of note, DC cultured in the presence of the anti-IL-8 mAb at 100 µg/mL did not show loss of viability at least in 72 h (data not shown). (B) Representative FACS dot plots from A in two tumor nodules from two mice are shown as an example. Similar data were obtained with xenografts of the CaCo2 cell line (Figure S2). (C) Absence of PKH26-labeled DC in 3 out of 3 SW48 xenografts processed as in A, following injection of fluorescence labeled human DC. In the left dot-plot, the fluorescence intensity of injected PKH26-labeled DC is shown for reference. Dot plots are from representative experiment of two actually performed with three animals per group each.

Human DC were derived from CD14^+^ monocytes in the presence of GM-CSF and IL-4 and labeled with PKH26. Fluorescent DC were injected into HT29 tumor nodules subcutaneously implanted into Rag^−/−^ IL-2Rγ^−/−^ mice. In some of the mice, DC were injected with control polyclonal mouse IgG antibody, while in other cases were resuspended with 100 µg/mL of an anti-human IL-8 neutralizing mAb. 72 h after DC injection, tumors were removed and a single cell suspension was generated. The number of fluorescent human CD11c^+^ cells versus total cells was quantified. As can be seen in figure 2A, IL-8 indeed retained DC intratumorally since the neutralizing anti-IL-8 mAb decreased the number of cells that remained inside the tumor by more than one half (Figure 2A). FACS analysis representing two cases are shown in figure 2B as an example. These results were confirmed in athymic nude mice bearing subcutaneous CaCo-2 tumors (Figure S3) indicating that the phenomenon was not exclusive of HT29-derived tumors.

SW48 cells, that failed to produce IL-8 as shown in figure S1, were xenografted in Rag^−/−^ mice. In this case, the tumors could not retain DC labeled with the fluorescent dye PKH26. Figure 2C shows representative dot plots from three mice 72 h post intratumoral injection along with the fluorescence of input DC (left dot-plot of figure 2C). These results on the colon cancer cell line that does not produce IL-8 further indicate that this chemokine was important for the retention of DC inside the tumor upon intratumoral release.

### Lack of IL-8 effects on DC-mediated T-cell stimulation

**Figure 3:**
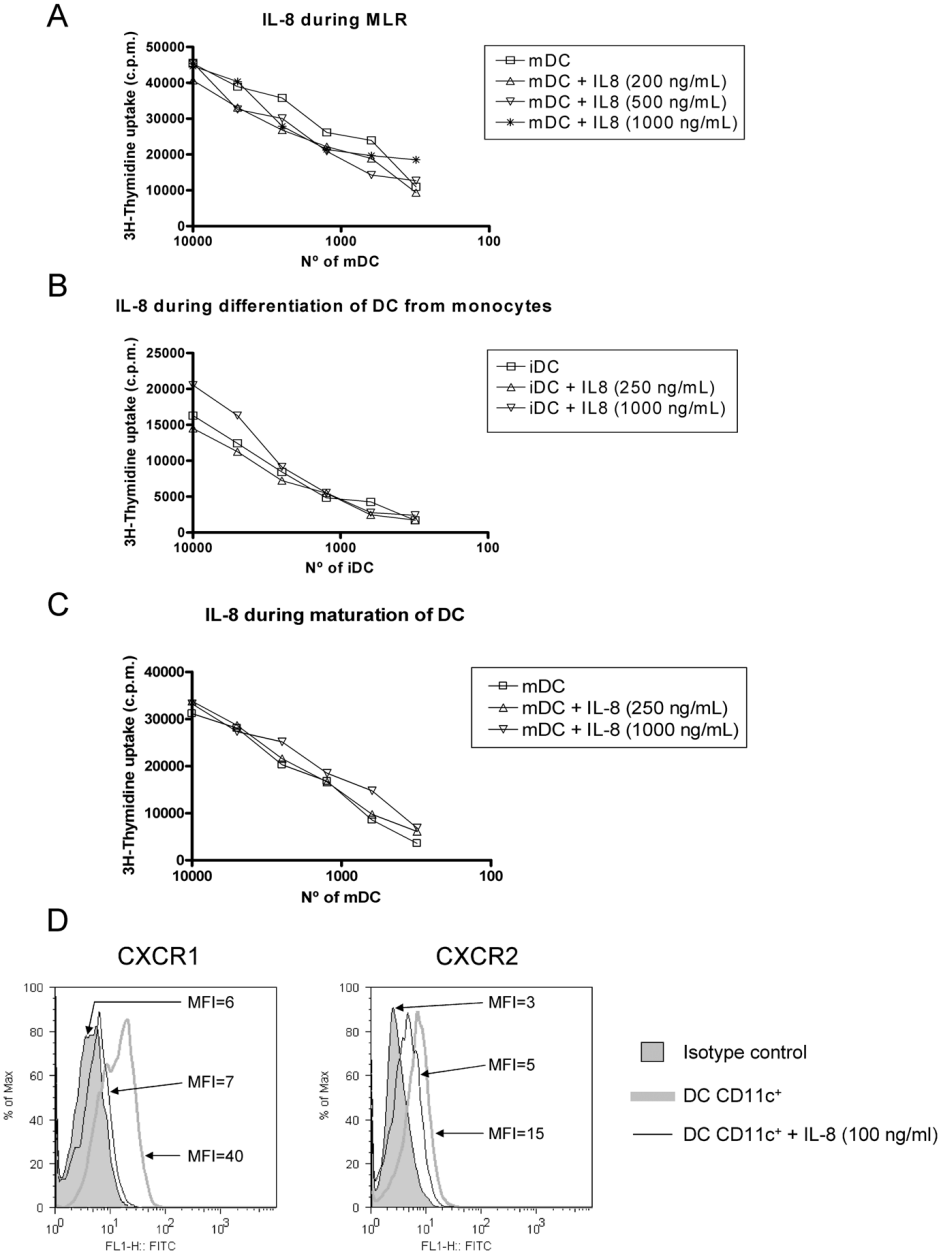
IL-8 does not impair T cell stimulation by DC that express functional CXCR1 and CXCR2. (A) Human monocyte-derived DC and T-cells were seeded in the indicated proportions. Functional recombinant IL-8 was added at different concentrations as indicated in the graph legends. T-cell proliferation was measured by ^3^H-thymidine incorporation 3 days later. A representative case out of three independently performed experiments with cells from different combinations of donors is shown. (B) Experiments as in A, but in this case DC were incubated with the indicated amounts of IL-8 added to monocytes during the 7-day differentiation culture in the presence of GM-CSF+IL-4. A representative experiment out of at least three is shown. (C) Similar experiments as in B but in this case IL-8 at the indicated concentrations was added during the maturation 48 h culture onto differentiated DC matured for 48 h with IFN-α, TNF-α and poly I:C. A representative experiment out of at least three performed is shown. As a control every IL-8 batch was shown to readily attract human PMNs in chemotaxis assays (Figure S3). (D) Mature DC as those used for the MLRs were tested by indirect immunofluorescence for the expression of CXCR1 and CXCR2. Incubation with 100 ng/mL of IL-8 during 2 h resulted in a loss of fluorescence intensity upon immunostaining of the surface receptors indicating receptor internalization. The mean fluorescence intensity (MFI) of each histogram is provided. FACS Experiments were repeated in two occasions with similar results.

Functional response to IL-8 involves signalling pathways that might alter DC functions, since these cells are known to express CXCR1 and CXCR2 [1]. We explored this question in detail using Mixed Lymphocyte Reaction (MLR) assays in which DC were co-cultured in decreasing amounts with fully allogeneic Peripheral Blood Mononuclear Cells (PBMC) containing alloreactive T-cells. When added during the MLR reaction, IL-8 did not change the proliferation of T cells (Figure 3A) or the ensuing production of INF-γ to the supernatant (data not shown).

DC were derived from CD14^+^ monocytes in the presence of GM-CSF and IL-4 and during this process we had observed that tumor-derived compounds impair differentiation [26]. However, in the case of IL-8 as a recombinant protein, resulting DC stimulated allogenic MLRs as strongly as those DC derived in the absence of the IL-8 chemokine (Figure 3B).

Alternatively, IL-8 could alter the maturation/activation of DC [29]. To induce maturation, DC were incubated for 48 h with a mixture of INF-α, TNF-α and Poly I:C in the presence or absence of IL-8. No change was observed again in the ability of DC to induce proliferation of allogeneic T lymphocytes (Figure 3C). To rule out alterations of the recombinant protein used, in every case, IL-8 was controlled for functionality since it readily attracted human neutrophils, as shown in chemotaxis assays (Figure S4).

We had previously shown that DC expressed CXCR1 and CXCR2 [1]. We observed that the exposure to the ligand for two hours induced the modulation/internalization of both receptors (Figure 3D) in the very same DC used to set up the T-cell allostimulation experiments. Therefore, the pathways guiding IL-8-directed migration and those governing the capabilities for T-cell stimulation seem to be fairly independent in the DC.

The absence of effects on T cell∶DC co-cultures suggested that the molecular factors employed by the DC for T cell activation were not affected by IL-8.

Indeed, table S1 shows that IL-8 at various concentrations does not alter the level of expression the maturation markers CD80, CD83, CD86 and MHC class II on mature and immature DC. Moreover, IL-8 did not alter the production of IL-12 and IL-10 upon maturation as induced by lipopolysaccharide (LPS) plus the R-848 imidazoquinoline or recombinant CD40L (Figure S5).

**Figure 4:**
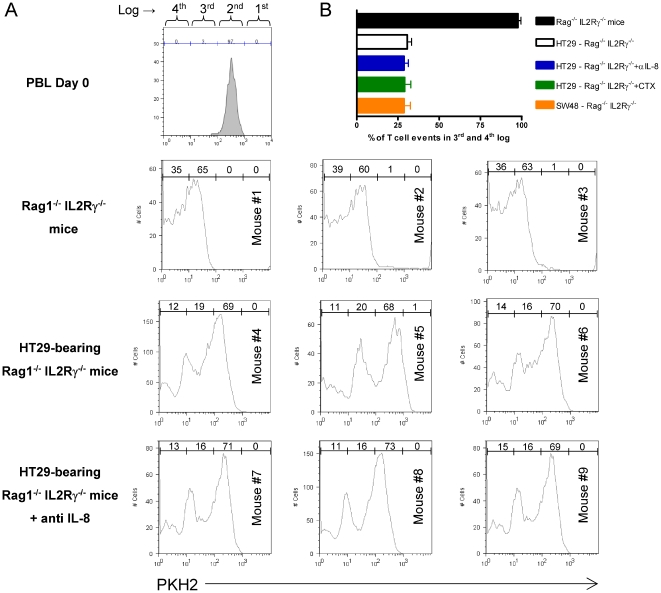
Impairment of DC-induced human T-cell proliferation inside the peritoneum of HT29 xenografted mice. (A) Rag^−/−^ IL-2γR^−/−^ mice (3 per group) were xenografted with HT29 cells or remained tumor-free. Four weeks later mice received intraperitoneal injections of human PKH2-labeled PBLs and fully allogenic mature DC (ratio 5∶1 to a total of 6×10^6^ cells). Proliferation was monitored four days later by dye dilution on FACS-gated lymphocytes from peritoneal lavages by dilution of the fluorescent dye. In the group of indicated mice an intraperitoneal injection of 100 µg of neutralizing anti-IL-8 mAb was provided immediately following the injection of PBLs and DC. Percentages of dividing lymphocytes in each log cursor interval are shown in the histograms. Fluorescence intensity in the input undivided PBL was over 95% above the third log interval (upper histogram). (B) Similar experiments as in A, quantifying PKH2-dilution as the percentage of cells that reach the 3^rd^ and 4^th^ log scales of the flow cytometry histograms. Log regions are depicted in the upper histogram of A. Data from animals bearing subcutaneous SW48 xenografts, that do not produce IL-8, have been included. Data represent mean±SD.

Furthermore, we explored the issue of whether immunodeficient animals bearing IL-8-producing tumors would impair MLR alloreactions of human leukocytes seeded inside their peritoneal cavities. For this purpose, we grafted HT29 tumors for 5–7 weeks and co-injected PKH2-labeled PBL and allogeneic DC inside the peritoneal cavity. As can be seen in figure 4, T cells readily proliferated in tumor-free mice within 4 days and such proliferative responses were clearly downsized by the presence of subcutaneous tumors.

If neutralizing anti-IL-8 mAb was co-injected with the PBL and the allogenic DC, no recovery of proliferation was observed. This is interpreted in the sense that factors other than IL-8 down-regulate T-cell proliferation. This is in agreement with the lack of IL-8 effects on DC-mediated T-cell stimulation in the *in vitro* alloreactive co-cultures.

In addition, we performed experiments (shown in figure 4B) that demonstrate that treatment of the HT29-xenografted mice with cyclophosphamide did not improve the alloreactive T-cell stimulation. Moreover SW48 xenografts, that do not produce IL-8, also inhibit the intraperitoneal alloreactive response to an extent comparable to that observed with HT29 (Figure 4B).

Regardless the fact that there is no IL-8 homologue gene in the mouse genome, human IL-8 exerts at least some agonist activity on mouse CXCR1 as described [6]. Indeed, we were able to observe IL-8 chemotactic activity on mouse bone marrow-derived DC (Figure S6A). Therefore we set up experiments in which we activated CD4^+^ TCR-transgenic OT-2 T cells responding to ovalbumin in the peritoneal cavity of Rag^−/−^ IL-2Rγ^−/−^ mice. DC were pulsed with the cognate peptide and the mice were bearing or not established HT29 subcutaneous tumors. As shown in figures S6B and C, HT29 tumors also suppressed proliferation of the mouse T cells in this setting, although such an inhibition was not affected again by IL-8 neutralizing antibodies. Our data further indicate the existence of immunosuppressive factors in the tumor bearing mice which are different from IL-8.

### Pre-exposure of DC to IL-8 disorient DC to subsequently follow IL-8-guided migration

DC in tumor bearing subjects would be chronically exposed to IL-8. As we have shown in figure 3D, exposure of DC to IL-8 determines receptor downregulation. Therefore we hypothesized that pre-incubation of DC with IL-8 could inhibit subsequent responses to IL-8 gradients.

**Figure 5:**
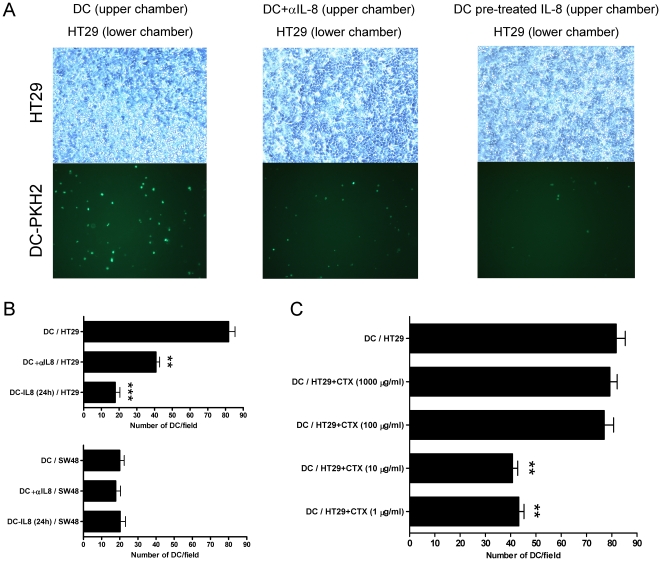
DC pre-exposed to IL-8 become desensitized to respond to carcinoma-derived IL-8 as a chemoattractant. (A) Chemotaxis assays were set up with HT29 confluent monolayers in the lower chamber and fluorescent DC in the upper chamber. Phase contrast microscopy images and the corresponding UV fluorescence microscopic fields of the lower chamber are shown. When indicated the lower chamber contained neutralizing anti-IL-8 mAb (20 µg/mL) or the DC had been pre-exposed for 24 h to recombinant IL-8 (1 µg/mL). (B) In the upper panel representation of data from three independent experiments similarly performed to those in A with HT29 cells represented as mean±SD in which four random fields were counted for each triplicate well. In the lower panel experiments performed as in A, but in this case the confluent monolayers in the lower chamber were formed by SW48 cells that do not produce IL-8. (C) Experiments as in A, but in this case HT29 cells had been pretreated with various cyclophosphamide concentrations for 24 h as indicated in the figure. Results represent mean±SD.

In figure 5, chemotactic experiments were set up plating IL-8-producing HT29 cells in the lower chamber and PKH2-labelled DC in the upper chamber. The colon carcinoma cell line induced migration of DC in 16 h that was abolished by anti-IL8 neutralizing mAb. Interestingly, if DC had been pre-exposed for 24 h to recombinant IL-8, migration was also abolished indicating that desensitized DC could not migrate towards the IL-8-producing carcinoma cells. Data are quantified in figure 5B, which also shows that monolayers of the SW48 (the cell line that does not produce IL-8) also fail to attract DC. Likewise, pre-treatment of the HT29 cells with low concentrations of cyclophosphamide decreased the ability of HT29 to attract DC because of reducing IL-8 secretion (Figure 5C).

Therefore while IL-8 seems to leave T cell stimulation by DC unimpaired, chronic exposure to IL-8 may profoundly affect the migration capabilities of DC towards IL-8 gradients and possibly of other leukocyte subsets as well.

In a previous study we reported that DC produce IL-8 [1]. Figure S7A confirms that DC produce IL-8 at the protein and mRNA level. It was conceivable the autocrine IL-8 may downregulate CXCR1 and CXCR2 expression, as seen in figure 3D with exogenously added IL-8. Indeed, we observed brighter immunofluorescence specific for CXCR1 and CXCR2 when IL-8 was neutralized with a specific mAb (Figures S7B and C) and when DC were cultured at very low cell densities upon agitation to dilute the secreted IL-8 (Figures S7B, C and D). Therefore autocrine IL-8 determines the level of receptor surface expression in DC providing an interesting mode of regulation.

### IL-8 produced by DC retains and attracts neutrophils

A function of IL-8 could be to favor a rendezvous between polymorphonuclear (PMN) cells and DC by co-attracting both subsets of leukocytes. In our hands, both immature and mature DC produce abundant IL-8, although mature DC produce about four-five-fold more quantity on a per cell basis (Figure S7).

**Figure 6:**
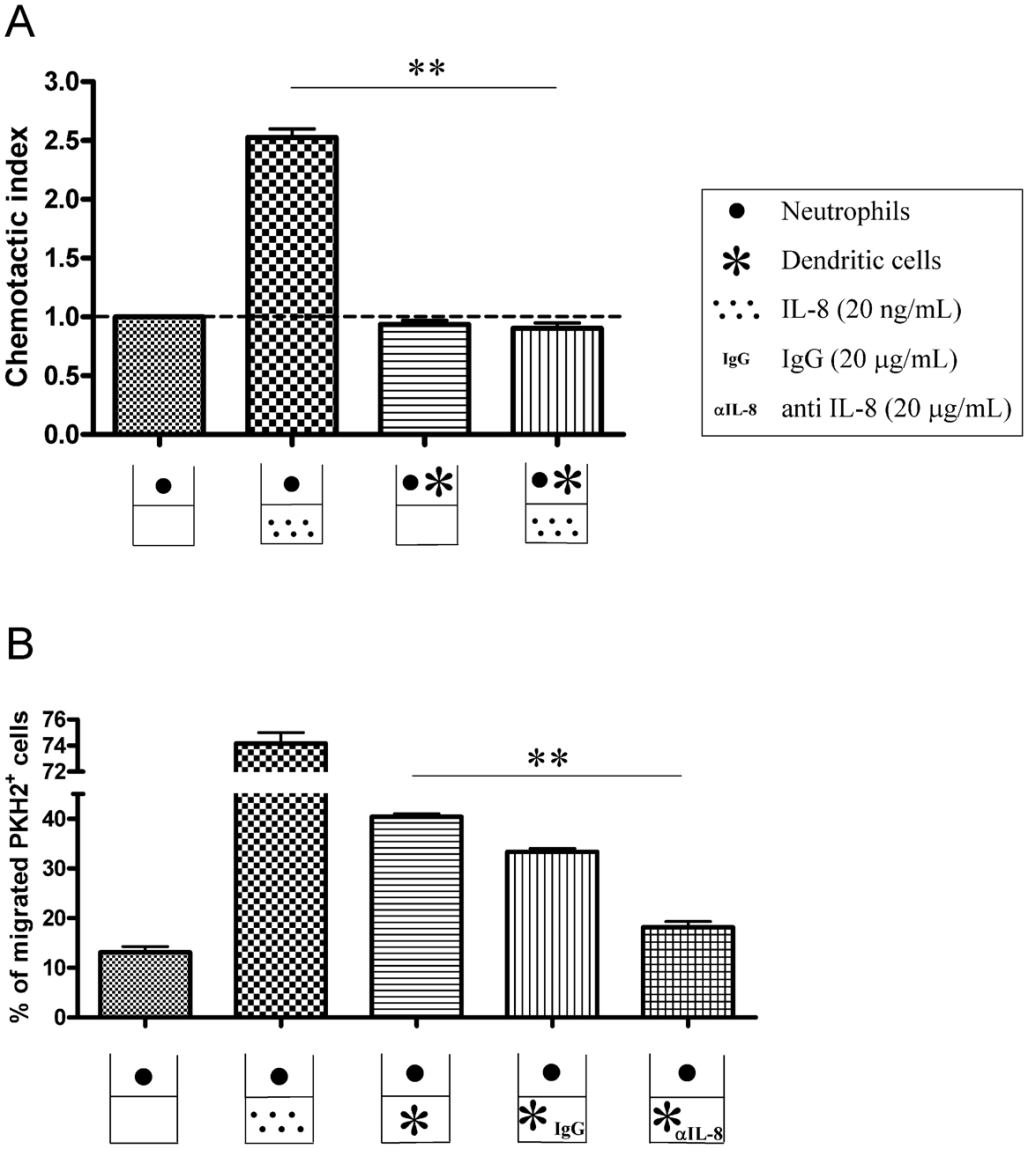
DCs retain neutrophils in migration assays towards IL-8 and attract neutrophils in an IL-8-dependent fashion. (A) Transwell chemotaxis assays were set up with PKH2-labeled neutrophils. Neutrophils migrated to recombinant IL-8 added to the lower chamber. However, if neutrophils are seeded in the upper chamber together with DC (1∶1) migration is totally impaired. Of note there is IL-8 by the DC as shown in figure S6A. This experiment is representative of two independently performed in triplicate wells with migration lasting for 120 min. (B) Percentage of migration of PKH2-labeled neutrophils that were seeded into the upper chamber of transwell migration assays towards recombinant IL-8 or DC placed in the lower chamber. When indicated, IL-8-neutralizing mAb (20 µg/mL) was added to the lower chamber along with the DC. Migration was analyzed at 120 minutes. Similar experiments analyzed as early as 60 minutes rendered similar results (data not shown). Results are representative of two separate triplicate experiments independently performed. Asterisks indicate statistical significance p<0.01 in student's t tests.

Classical chemotaxis assays were set up to determine if IL8 could regulate DC and PMN migration in a concerted fashion. As can be seen in figure 6A and figure S4, neutrophils are attracted by recombinant IL-8. However, if neutrophils are seeded together with DC (at 1∶1 ratio), neutrophil migration as induced by IL-8 was abolished. These data might indicate that DC have a means to attract and/or retain PMNs that otherwise would migrate away.

If the assays were set up with PMNs in the upper and DC in the lower chamber, neutrophils were attracted by DC seeded into the lower chamber. Importantly, addition of neutralizing anti-IL-8 mAb eliminated most of the attraction of fluorescence-labeled neutrophils by the DC seeded in the lower chamber (Figure 6B) while control antibody exerted no effect.

Our results as a whole indicate that although IL-8, abundantly produced by tumors, would not damage DC-mediated stimulation of T-cells. However, tumor-derived IL-8 would alter migration and interactions with other leukocyte subtypes such as neutrophils. For instance, if DC are prevented from migrating towards MIP3α gradients by HT29 supernatants they would reach the inflammatory focus in lesser numbers and as a result would stimulate T cells less efficiently (Figure S8). IL-8-disoriented migration could thereby contribute to weaken immune responses to cancer.

### IL-8 produced by tumor xenografts attracts DC to the tumor tissue unless they have been desensitized by pre-exposure to IL-8

**Figure 7:**
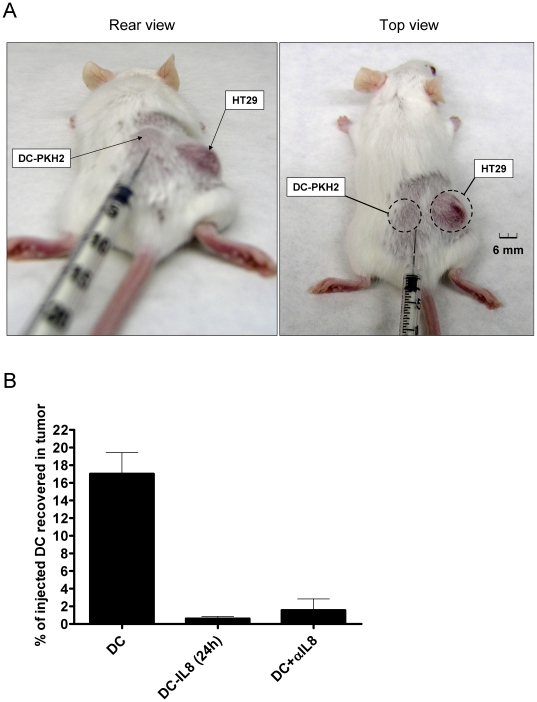
HT29 xenografts attract human DC injected in the subcutaneous tissue which surrounds the tumor. (A) Mice bearing HT29 established xenografts as the one shown in the pictures were injected approximately 5 mm away from the tumor lesion with 5×10^6^ human immature DC labelled with PKH2 that were resuspended in 50 µl of saline buffer to form a small subcutaneous bump which disappeared in less than 2 hours. (B) 24 hours later tumors were surgically removed and a cell suspension was obtained in which the number of fluorescent DC were enumerated by flow cytometry and normalized as the percentage of injected DC that were recovered from the tumor. When indicated DC were pre-treated for 24 hours in culture with 1 µg/ml of rIL-8 or DC were co-injected with 100 µg of neutralizing anti-IL8 mAb.

As seen in figure 1, HT29 xenografts are sites of intense IL-8 production. Therefore we reasoned that DC injected in the subcutaneous tissue 5 mm away from the tumor (Figure 7A) should be attracted to the tumor nodule. Indeed, fluorescence-labelled DC were recovered from the tumor tissue and such migratory behaviour was inhibited if DC were co-injected with the neutralizing anti-IL-8 mAb (Figure 7B). More importantly, pre-exposure of the DC cultures to IL-8 for 24 h prior to injection also greatly impaired the migration towards the tumor. Therefore tumors can attract DC by means of IL-8 but chronic exposure to IL-8 desensitizes DC for this in vivo migration. Owing to these effects, IL-8 produced at malignant lesions profoundly impairs the migratory orientation of DC.

## Discussion

By producing IL-8, tumors may profoundly alter the migration-guiding gradients of this important chemokine in the tissues of tumor-bearing hosts [33]. Indeed, the chemokine network is well known to modify cancer biology in multiple ways from metastasis and angiogenesis to the attraction of a nurturing leukocyte infiltrates [33]. In this study, we demonstrate that IL-8 as produced by human tumor cells is capable of attracting (or retaining) human DC *in vivo*, but that IL-8 does not functionally affect the ability of DC to stimulate alloreactive T cells.

We found that at least *in vitro*, IL-8 production by tumor cells can be decreased by low dose cyclophosphamide at the protein and mRNA level. *In vivo* this is reflected by transient decreases in the serum concentration of the chemokine in circulating blood. The rapid decrease and recovery of serum concentrations indicate the rapid turnover in blood of a polypeptide below the renal filtration threshold and with a short half-life [34]. We are exploring the therapeutic implications of low-dose cyclophosphamide effects on IL-8 patho-phisiology. In fact, this effects on IL-8 output can be a factor behind the beneficial effects of the described metronomic dosing of the drug [31], [32]. In addition acute reductions in IL-8 output as induced by cyclophosphamide might be exploited to support intratumoral injections of DC in order to favor migration to lymph nodes.

In a clinical trial IL-8 was suspected to mediate intratumoral retention of DC artificially delivered in such locations by image-guided procedures of injection [1]. However, the role of IL-8 at *in vivo* retention could not be experimentally documented. In this study, we observe that DC are retained inside xenografted colon carcinomas by IL-8. The evidence was generated by means of a neutralizing anti-IL-8 mAb injected alongside the DC. These findings further support our interpretations with regard to the apparent retention of intratumorally injected DC in patients according to scintigraphic scans [1], [2].

In our mouse xenograft system, we do not yet understand whether migration out of tumors is the result of chemotaxis driven by mouse factors or random migration. What we document is that IL-8 mediates the retention inside the tumor microenvironment. This is not a surprise since IL-8 receptors, CXCR1 and CXCR2, are expressed on DC and are functional in classical chemotaxis assays [1]. The production of IL-8 by DC themselves could be also operating in an autocrine or paracrine fashion in this intratumoral setting. Nonetheless, we clearly observe that HT29 tumor xenografts are capable to chemoattract DC when injected in the connective tissue that surrounds the malignancy.

The IL-8 receptors, when ligated, turn on various signaling pathways [35] and rearrange the cytoskeleton [13]. IL-8 could thereby potentially alter the functional performance of DC. Therefore we hypothesized that DC under the influence of IL-8 in the tumor, would be poorer T cell stimulators. However, fully functional IL-8 (as checked in migration assays) was completely incapable of decreasing T-cell allostimulation as mediated by DC. This is in agreement with the fact that IL-8 does not affect the expression of costimulatory receptors or T-cell stimulating cytokines. It remains to be seen if IL-8 alters the antigen presenting machinery or other biological activities of DC that are not required for alloreactive stimulation. Although not formally ruled out, this possibility is considered unlikely.

Nonetheless, if DC were retained in the tumor milieu by IL-8, those DC would remain under the concentrated influence of tumor-derived factors that repress DC functions [26], [36], [37], [38]. Evidence for this phenomenon also comes out from our alloreactive reactions of human lymphocytes inside the peritoneum of immunodeficient mice bearing HT29 and SW48 xenografts [39]. Some of the malignant tissue immunorepressor molecules include TGF-β [40], [41], VEGF [42], [43], interleukin-13 [44], prostaglandins, kynurerines [40] and most likely other unknown polypeptide moieties [26], as well as certain lipids [45].

Collectively, our data can be interpreted in the sense that IL-8 retains DC in the precise location where such antigen presenting cells are most efficiently damaged in their function by tumor-derived biomolecules. Our results regarding the *in vivo* inhibition of T-cell allostimulation in animals xenografted with human tumors offer a useful experimental system to dissect tumor-dependent mechanisms of inhibition that are operating distantly from the tumor implant. This experimental tool is employed in our laboratory [39].

DC intratumoral retention has been described as an evasion strategy in breast cancers [46]. We can observe in *in vitro* chemotaxis that if HT29 cells prevent DC from migrating to MIP3α mimicking an inflammatory focus then fewer DC reach the location to stimulate T cells and the immune response is accordingly less intense.

These phenomena certainly pose an obstacle for the intratumoral route of DC administration in human immunotherapy [11], an approach that has been described to be very successful in a number mouse models [47], [48], [49], [50]. From the therapeutic point of view, low doses of cyclophosphamide can be useful to reduce IL-8 output by viable tumor cells. Neutralization of IL-8 with mAb could be also therapeutically feasible. This has been recently demonstrated in a situation in which tumor xenografts escape from sunitinib-induced anti-angiogenesis by means of an IL-8-dependent mechanism [19].

IL-8 attracts neutrophils and possibly immature forms such as myeloid derived suppressor cells [37], [51]. Interestingly, DC secrete IL-8 that may act in an autocrine fashion [1]. Apart from these largely unexplored autocrine effects that modulate CXCR1 and CXCR2 surface levels; we show that DC are capable of attracting or retaining neutrophils in an IL-8-dependent fashion. The physiological consequences of DC-neutrophil interactions [52] in the tumor context are currently being actively explored in our laboratory, with emphasis on the implications for cross-presentation of tumor antigens [53], [54].

What seems also plausible is that under high circulating levels of IL-8, as occurs in HT29-xenografted mice or patients with bulky disease, migration-driving gradients of IL-8 would be disrupted and thereby DC-production of IL-8 might be overwhelmed in its ability to attract neutrophils and other leukocytes. Indeed, we have obtained evidence in the sense that overwhelming pre-exposure to IL-8 results in desensitization of the DC to the chemotactic effects of IL-8. In other words, this could mean that DC chronically exposed to IL-8 in the context of tumor-bearing hosts would become disoriented and thus unable to follow migration cues set up by IL-8 concentration gradients. *In vivo* evidence of desensitization for migration further supports this notion. Such immune disorientation as caused by the abundantly and ectopically expressed chemokine may result in disordered immune responses and ought to have relevant prognostic consequences for patients.

## Supporting Information

Figure S1 **Colon carcinoma cell lines HT29 and CaCo2 produce high levels of IL-8 in a clonal stable fashion while SW48 does not produce IL-8.** Clonal limiting dilution subcultures (four for each cell line) of the colon cancer-derived cell lines HT29, CaCo2 and SW48 were tested for the production of IL-8 as measured in 24 h culture supernatants by ELISA.
(TIF)

Figure S2 **CaCo2 carcinoma cells xenografted in immunodeficient mice develop progressive intraperitoneal tumors that correlate with raising serum concentrations of IL-8.** Intraperitoneally xenografted CaCo2 cells developed progressive peritoneal colon carcinomas in athymic nude mice (measured as weight increase in the left axis) and accumulated increasing concentrations of serum IL-8 (right axis).
(TIF)

Figure S3 **DC are retained inside CaCo2 tumors in a IL-8-dependent fashion.** CaCo2 cells were xenografted in athymic nude mice. Only 1/3 of such animals successfully xenografted tumor lesions. Tumor nodules, 8–12 mm in diameter, were injected with CFSE-labeled human DC derived from monocytes, as in A. DC were injected in 100 µL of saline buffer with control antibody or neutralizing anti-IL-8 mAb. In these cases, tumors were homogenated and cleared of debris by centrifugation. Fluorescence in the lysate was measured in a fluorimeter. The amount of fluorescence remaining in the tumor compared to that present in the lysate from an identical number of DC before being injected in the tumor was quantitated. Data are presented as the percentage of fluorescence lost from the tumor. Experiments were performed with four mice bearing a single tumor nodule. The inset shows a correlation of fluorescence (arbitrary units) and number of DC in lysates containing increasing amounts of CFSE-labeled DC.
(TIF)

Figure S4 **Recombinant IL-8 rendering negative results at modifying DC functionality is capable of attracting PMNs.** Migration transwell assays with purified neutrophils in the upper chamber and different concentrations of the recombinant IL-8 in the lower chamber to prove that IL-8 used in figure 3A, B, C and D was fully functional.
(TIF)

Figure S5 **IL-8 does not modify IL-12 nor IL-10 secretion by DC matured in the presence of LPS+R848 or trimerized CD40L.** IL-12 and IL-10 were quantified in the supernatant of DC cultures treated with the indicated concentrations of IL-8 during the 48 h maturation culture. Concentrations (mean±SD) represent triplicate wells from a single experiment.
(TIF)

Figure S6 **Activation of antigen specific murine CD4 T cells by DC in mice bearing HT29 tumors is suppressed by factors distinct from IL-8.** (A) Mouse BM-derived DC [50], [55] were subjected to classical transwell chemotaxis assays towards culture medium (control), 10^5^ heat-inactivated E. coli bacteria used as a positive control, or recombinant IL-8 as indicated. Data show a modest but reproducible attraction of mouse DC by human recombinant IL-8. (B) Schematic representation of experiments in which HT29-bearing Rag^−/−^ IL-2Rγ^−/−^ mice were injected in the peritoneal cavity with 5×10^6^ CFSE-labelled CD4 OT-2 cells [56] and 10^6^ syngeneic DC pulsed with the OVA323-339 synthetic peptide. (C) Assessment of OT-2 T-cell proliferation by dilution of CFSE as in figure 4. Experiments were performed in mice bearing or not HT29 tumors with or without cognate peptide stimulation by the DC (n = 3 mice per group). When indicated 100 µg of anti-IL8 mAb were co-injected into the peritoneal cavity.
(TIF)

Figure S7 **DC produce IL-8 and such autocrine IL-8 modulates in part the surface expression of CXCR1 and CXCR2.** (A) Left axis: IL-8 concentration in the supernatant of mature (mDC) and immature DC (iDC); Right axis: mRNA encoding IL-8 in the corresponding DC cultures assessed by semi quantitative RT-PCR. (B and C) IL-8 concentration in the supernatant (left axes) and CXCR1 (B) and CXCR2 (C) surface expression as mean fluorescence intensity (MFI) analyzed by FACS (right axes). In B and C a mAb neutralising IL-8 (20 µg/ml) was added when indicated, or the DC were cultured under gentle agitation at the cellular densities given. Results are presented as mean±SD from triplicate experiments. IL-8 neutralisation or lower IL8 concentrations in the supernatants correlate with higher MFIs for CXCR1 and CXCR2 on the DC. (D) Shows representative FACS histograms from B and C.
(TIF)

Figure S8 **IL-8 in conditioned supernatants from HT29 cells impedes DC from migrating to MIP3α gradients.** As a consequence fewer DC reaching the lower chamber results in less T-cell allostimulatory activity at this location. In order to model whether DC-disoriented migration would give rise to less T cell stimulation, we set up in the left panel chemotaxis assays in which DC migrated towards recombinant MIP3α (100 µg/ml). Data are presented as mean±SD of the chemotactic index normalized with culture medium without MIP3α (Neg). DC were seeded in the upper chamber with or without conditioned medium of HT29 cells or SW48 cells as indicated. When indicated an IL-8 neutralising antibody was added. In the right panel DC recovered from the lower chamber were used to stimulate allogenic PBL, and T-cell proliferation was recorded three days later as c.p.m. in ^3^H-Thy incorporation assays. Data represent three independent replicates.
(TIF)

Table S1 **Lack of IL-8 effect on surface expression of dendritic cell maturation markers.** Mean fluorescence intensity of the indicated surface markers of DC upon FACS analyses in human DC (mean±SD from three different experiments) using either immature (iDC) or LPS+R848 matured DC (mDC), that were cultured in the absence or the presence of increasing concentrations of IL-8 as indicated in the columns. Experiments are representative of three similarly performed with different donors.
(TIF)

## References

[1] Feijoo E, Alfaro C, Mazzolini G, Serra P, Penuelas I (2005). Dendritic cells delivered inside human carcinomas are sequestered
by interleukin-8.. Int J Cancer.

[2] Mazzolini G, Alfaro C, Sangro B, Feijoo E, Ruiz J (2005). Intratumoral injection of dendritic cells engineered to secrete
interleukin-12 by recombinant adenovirus in patients with metastatic
gastrointestinal carcinomas.. J Clin Oncol.

[3] Xie K (2001). Interleukin-8 and human cancer biology.. Cytokine Growth Factor Rev.

[4] Sallusto F, Palermo B, Lenig D, Miettinen M, Matikainen S (1999). Distinct patterns and kinetics of chemokine production regulate
dendritic cell function.. Eur J Immunol.

[5] Sozzani S, Luini W, Borsatti A, Polentarutti N, Zhou D (1997). Receptor expression and responsiveness of human dendritic cells
to a defined set of CC and CXC chemokines.. J Immunol.

[6] Fan X, Patera AC, Pong-Kennedy A, Deno G, Gonsiorek W (2007). Murine CXCR1 is a functional receptor for GCP-2/CXCL6 and
interleukin-8/CXCL8.. J Biol Chem.

[7] Sallusto F, Lanzavecchia A (1999). Mobilizing dendritic cells for tolerance, priming, and chronic
inflammation.. J Exp Med.

[8] Martin-Fontecha A, Lanzavecchia A, Sallusto F (2009). Dendritic cell migration to peripheral lymph
nodes.. Handb Exp Pharmacol.

[9] MartIn-Fontecha A, Sebastiani S, Hopken UE, Uguccioni M, Lipp M (2003). Regulation of dendritic cell migration to the draining lymph
node: impact on T lymphocyte traffic and priming.. J Exp Med.

[10] Verdijk P, Aarntzen EH, Punt CJ, de Vries IJ, Figdor CG (2008). Maximizing dendritic cell migration in cancer
immunotherapy.. Expert Opin Biol Ther.

[11] Huarte E, Tirapu I, Arina A, Vera M, Alfaro C (2005). Intratumoural administration of dendritic cells: hostile
environment and help by gene therapy.. Expert Opin Biol Ther.

[12] Bachmann MF, Kopf M, Marsland BJ (2006). Chemokines: more than just road signs.. Nat Rev Immunol.

[13] Waugh DJ, Wilson C (2008). The interleukin-8 pathway in cancer.. Clin Cancer Res.

[14] Murdoch C, Muthana M, Coffelt SB, Lewis CE (2008). The role of myeloid cells in the promotion of tumour
angiogenesis.. Nat Rev Cancer.

[15] Schraufstatter IU, Trieu K, Zhao M, Rose DM, Terkeltaub RA (2003). IL-8-mediated cell migration in endothelial cells depends on
cathepsin B activity and transactivation of the epidermal growth factor
receptor.. J Immunol.

[16] Lin WW, Karin M (2007). A cytokine-mediated link between innate immunity, inflammation,
and cancer.. J Clin Invest.

[17] Rolny C, Capparuccia L, Casazza A, Mazzone M, Vallario A (2008). The tumor suppressor semaphorin 3B triggers a prometastatic
program mediated by interleukin 8 and the tumor
microenvironment.. J Exp Med.

[18] Ueda T, Shimada E, Urakawa T (1994). Serum levels of cytokines in patients with colorectal cancer:
possible involvement of interleukin-6 and interleukin-8 in hematogenous
metastasis.. J Gastroenterol.

[19] Huang D, Ding Y, Zhou M, Rini BI, Petillo D Interleukin-8 mediates resistance to antiangiogenic agent
sunitinib in renal cell carcinoma.. Cancer Res.

[20] Baggiolini M, Moser B, Clark-Lewis I (1994). Interleukin-8 and related chemotactic cytokines. The Giles Filley
Lecture.. Chest.

[21] Baggiolini M, Loetscher P (2000). Chemokines in inflammation and immunity.. Immunol Today.

[22] Stillie R, Farooq SM, Gordon JR, Stadnyk AW (2009). The functional significance behind expressing two IL-8 receptor
types on PMN.. J Leukoc Biol.

[23] Melero I, Vile RG, Colombo MP (2000). Feeding dendritic cells with tumor antigens: self-service buffet
or a la carte?. Gene Ther.

[24] Guo J, Zhu J, Sheng X, Wang X, Qu L (2007). Intratumoral injection of dendritic cells in combination with
local hyperthermia induces systemic antitumor effect in patients with
advanced melanoma.. Int J Cancer.

[25] Triozzi PL, Khurram R, Aldrich WA, Walker MJ, Kim JA (2000). Intratumoral injection of dendritic cells derived in vitro in
patients with metastatic cancer.. Cancer.

[26] Alfaro C, Suarez N, Gonzalez A, Solano S, Erro L (2009). Influence of bevacizumab, sunitinib and sorafenib as single
agents or in combination on the inhibitory effects of VEGF on human
dendritic cell differentiation from monocytes.. Br J Cancer.

[27] Verdijk P, Aarntzen EH, Lesterhuis WJ, Boullart AC, Kok E (2009). Limited amounts of dendritic cells migrate into the T-cell area
of lymph nodes but have high immune activating potential in melanoma
patients.. Clin Cancer Res.

[28] de Vries IJ, Lesterhuis WJ, Barentsz JO, Verdijk P, van Krieken JH (2005). Magnetic resonance tracking of dendritic cells in melanoma
patients for monitoring of cellular therapy.. Nat Biotechnol.

[29] Melief CJ (2008). Cancer immunotherapy by dendritic cells.. Immunity.

[30] Meyer TP, Zehnter I, Hofmann B, Zaisserer J, Burkhart J (2005). Filter Buffy Coats (FBC): a source of peripheral blood leukocytes
recovered from leukocyte depletion filters.. J Immunol Methods.

[31] Gasparini G (2001). Metronomic scheduling: the future of
chemotherapy?. Lancet Oncol.

[32] Ghiringhelli F, Menard C, Puig PE, Ladoire S, Roux S (2007). Metronomic cyclophosphamide regimen selectively depletes
CD4+CD25+ regulatory T cells and restores T and NK effector
functions in end stage cancer patients.. Cancer Immunol Immunother.

[33] Balkwill F (2004). Cancer and the chemokine network.. Nat Rev Cancer.

[34] Gross MD, Shapiro B, Fig LM, Steventon R, Skinner RW (2001). Imaging of human infection with (131)I-labeled recombinant human
interleukin-8.. J Nucl Med.

[35] Rossi D, Zlotnik A (2000). The biology of chemokines and their receptors.. Annu Rev Immunol.

[36] Tirapu I, Huarte E, Guiducci C, Arina A, Zaratiegui M (2006). Low surface expression of B7-1 (CD80) is an immunoescape
mechanism of colon carcinoma.. Cancer Res.

[37] Rabinovich GA, Gabrilovich D, Sotomayor EM (2007). Immunosuppressive strategies that are mediated by tumor
cells.. Annu Rev Immunol.

[38] Zou W, Chen L (2008). Inhibitory B7-family molecules in the tumour
microenvironment.. Nat Rev Immunol.

[39] Suarez N, Alfaro C, Dubrot J, Palazon A, Bolanos E Synergistic effects of CTLA-4 blockade with tremelimumab and
elimination of regulatory T lymphocytes in vitro and in
vivo.. Int J Cancer.

[40] Belladonna ML, Orabona C, Grohmann U, Puccetti P (2009). TGF-beta and kynurenines as the key to infectious
tolerance.. Trends Mol Med.

[41] Rutella S, Danese S, Leone G (2006). Tolerogenic dendritic cells: cytokine modulation comes of
age.. Blood.

[42] Conejo-Garcia JR, Benencia F, Courreges MC, Kang E, Mohamed-Hadley A (2004). Tumor-infiltrating dendritic cell precursors recruited by a
beta-defensin contribute to vasculogenesis under the influence of
Vegf-A.. Nat Med.

[43] Gabrilovich DI, Chen HL, Girgis KR, Cunningham HT, Meny GM (1996). Production of vascular endothelial growth factor by human tumors
inhibits the functional maturation of dendritic cells.. Nat Med.

[44] Aspord C, Pedroza-Gonzalez A, Gallegos M, Tindle S, Burton EC (2007). Breast cancer instructs dendritic cells to prime interleukin
13-secreting CD4+ T cells that facilitate tumor
development.. J Exp Med.

[45] Herber DL, Cao W, Nefedova Y, Novitskiy SV, Nagaraj S Lipid accumulation and dendritic cell dysfunction in
cancer.. Nat Med.

[46] Bell D, Chomarat P, Broyles D, Netto G, Harb GM (1999). In breast carcinoma tissue, immature dendritic cells reside
within the tumor, whereas mature dendritic cells are located in peritumoral
areas.. J Exp Med.

[47] Kikuchi T, Moore MA, Crystal RG (2000). Dendritic cells modified to express CD40 ligand elicit
therapeutic immunity against preexisting murine tumors.. Blood.

[48] Miller PW, Sharma S, Stolina M, Butterfield LH, Luo J (2000). Intratumoral administration of adenoviral interleukin 7
gene-modified dendritic cells augments specific antitumor immunity and
achieves tumor eradication.. Hum Gene Ther.

[49] Nishioka Y, Hirao M, Robbins PD, Lotze MT, Tahara H (1999). Induction of systemic and therapeutic antitumor immunity using
intratumoral injection of dendritic cells genetically modified to express
interleukin 12.. Cancer Res.

[50] Tirapu I, Arina A, Mazzolini G, Duarte M, Alfaro C (2004). Improving efficacy of interleukin-12-transfected dendritic cells
injected into murine colon cancer with anti-CD137 monoclonal antibodies and
alloantigens.. Int J Cancer.

[51] Gabrilovich DI, Nagaraj S (2009). Myeloid-derived suppressor cells as regulators of the immune
system.. Nat Rev Immunol.

[52] van Gisbergen KP, Sanchez-Hernandez M, Geijtenbeek TB, van Kooyk Y (2005). Neutrophils mediate immune modulation of dendritic cells through
glycosylation-dependent interactions between Mac-1 and
DC-SIGN.. J Exp Med.

[53] Melero I, Arina A, Murillo O, Dubrot J, Alfaro C (2006). Immunogenic cell death and cross-priming are reaching the
clinical immunotherapy arena.. Clin Cancer Res.

[54] Murillo O, Dubrot J, Palazon A, Arina A, Azpilikueta A (2009). In vivo depletion of DC impairs the anti-tumor effect of
agonistic anti-CD137 mAb.. Eur J Immunol.

[55] Melero I, Duarte M, Ruiz J, Sangro B, Galofre J (1999). Intratumoral injection of bone-marrow derived dendritic cells
engineered to produce interleukin-12 induces complete regression of
established murine transplantable colon adenocarcinomas.. Gene Ther.

[56] Barnden MJ, Allison J, Heath WR, Carbone FR (1998). Defective TCR expression in transgenic mice constructed using
cDNA-based alpha- and beta-chain genes under the control of heterologous
regulatory elements.. Immunol Cell Biol.

