# Supplementary figures and images for: Carcinoma-Derived Interleukin-8 Disorients Dendritic Cell Migration Without Impairing T-Cell Stimulation
Source: PLoS One. 2011 Mar 14;6(3):e17922. doi: 10.1371/journal.pone.0017922 (PMC3056721; doi:10.1371/journal.pone.0017922)

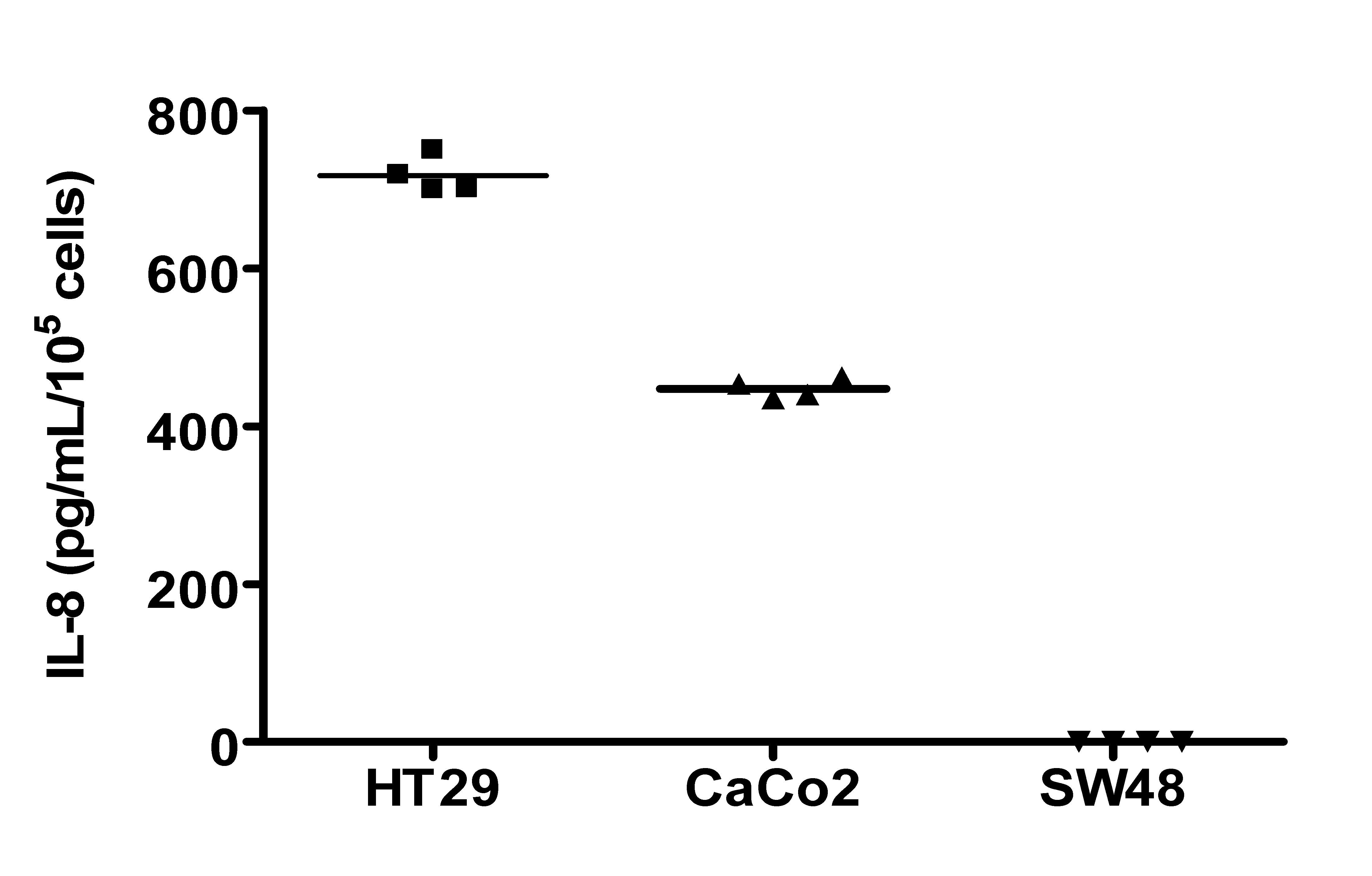

Supplement: Figure S1 — Colon carcinoma cell lines HT29 and CaCo2 produce high levels of IL-8 in a clonal stable fashion while SW48 does not produce IL-8. Clonal limiting dilution subcultures (four for each cell line) of the colon cancer-derived cell lines HT29, CaCo2 and SW48 were tested for the production of IL-8 as measured in 24 h culture supernatants by ELISA. (TIF) [file pone.0017922.s001.tif]

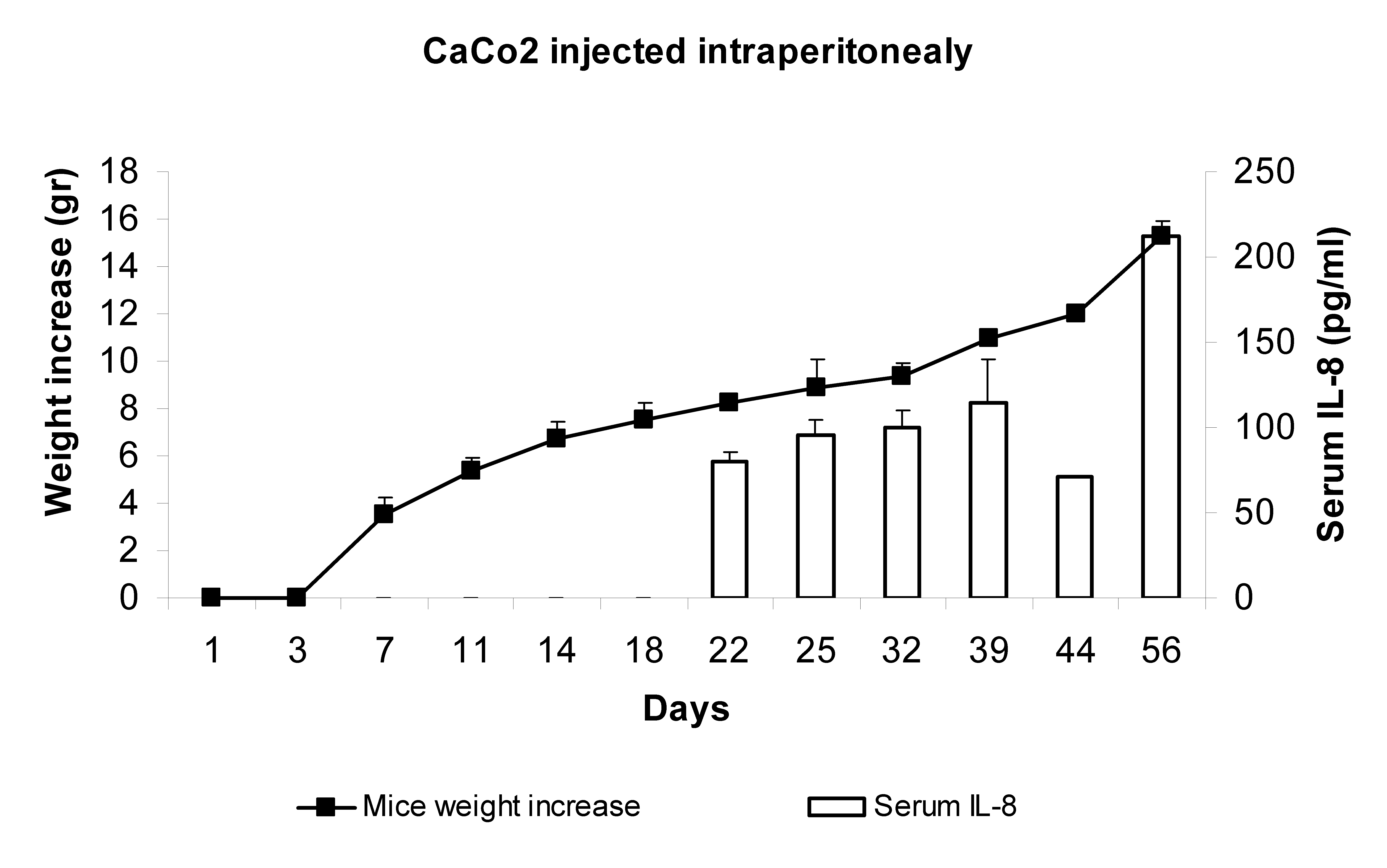

Supplement: Figure S2 — CaCo2 carcinoma cells xenografted in immunodeficient mice develop progressive intraperitoneal tumors that correlate with raising serum concentrations of IL-8. Intraperitoneally xenografted CaCo2 cells developed progressive peritoneal colon carcinomas in athymic nude mice (measured as weight increase in the left axis) and accumulated increasing concentrations of serum IL-8 (right axis). (TIF) [file pone.0017922.s002.tif]

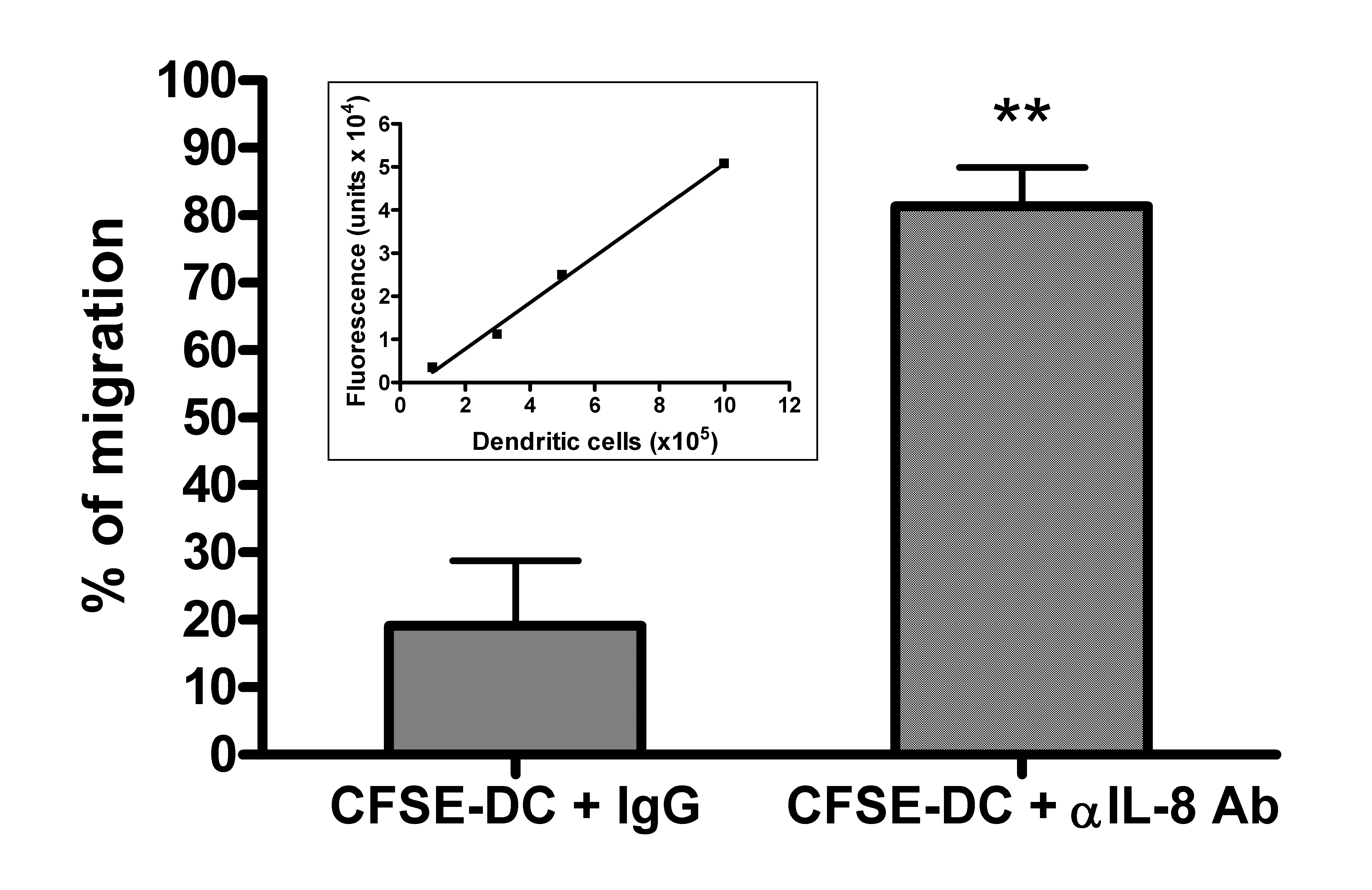

Supplement: Figure S3 — DC are retained inside CaCo2 tumors in a IL-8-dependent fashion. CaCo2 cells were xenografted in athymic nude mice. Only 1/3 of such animals successfully xenografted tumor lesions. Tumor nodules, 8–12 mm in diameter, were injected with CFSE-labeled human DC derived from monocytes, as in A. DC were injected in 100 µL of saline buffer with control antibody or neutralizing anti-IL-8 mAb. In these cases, tumors were homogenated and cleared of debris by centrifugation. Fluorescence in the lysate was measured in a fluorimeter. The amount of fluorescence remaining in the tumor compared to that present in the lysate from an identical number of DC before being injected in the tumor was quantitated. Data are presented as the percentage of fluorescence lost from the tumor. Experiments were performed with four mice bearing a single tumor nodule. The inset shows a correlation of fluorescence (arbitrary units) and number of DC in lysates containing increasing amounts of CFSE-labeled DC. (TIF) [file pone.0017922.s003.tif]

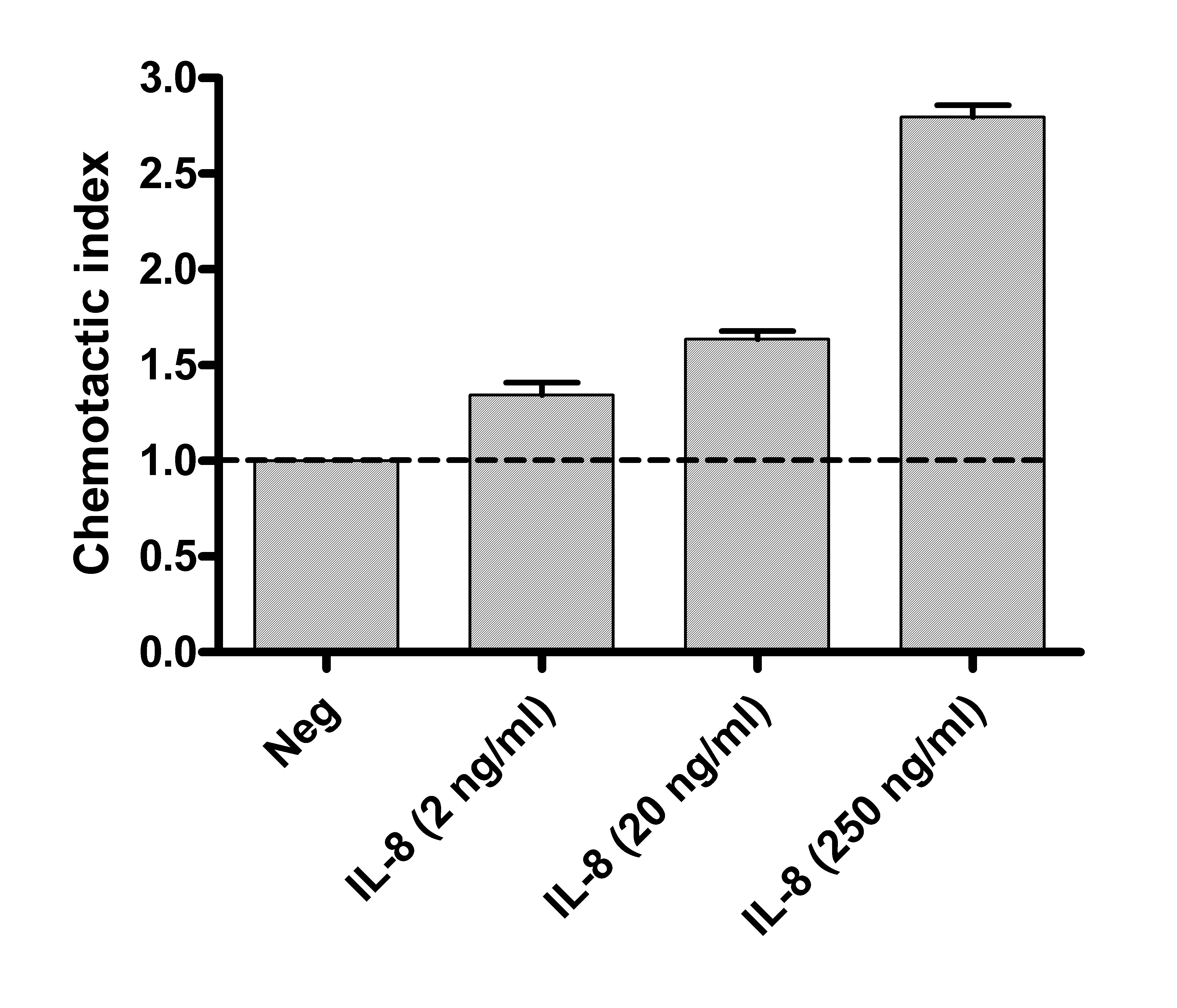

Supplement: Figure S4 — Recombinant IL-8 rendering negative results at modifying DC functionality is capable of attracting PMNs. Migration transwell assays with purified neutrophils in the upper chamber and different concentrations of the recombinant IL-8 in the lower chamber to prove that IL-8 used in figure 3A, B, C and D was fully functional. (TIF) [file pone.0017922.s004.tif]

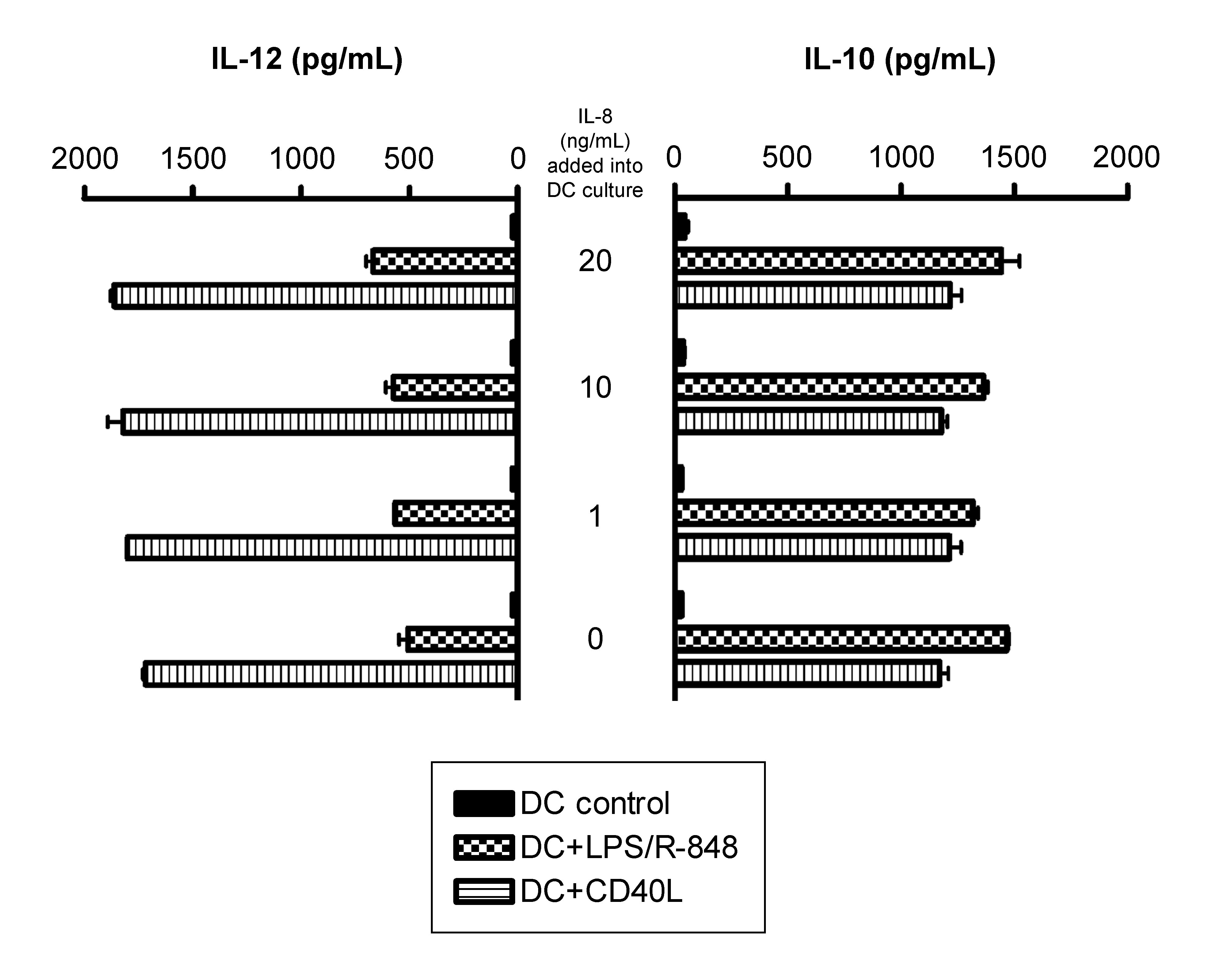

Supplement: Figure S5 — IL-8 does not modify IL-12 nor IL-10 secretion by DC matured in the presence of LPS+R848 or trimerized CD40L. IL-12 and IL-10 were quantified in the supernatant of DC cultures treated with the indicated concentrations of IL-8 during the 48 h maturation culture. Concentrations (mean±SD) represent triplicate wells from a single experiment. (TIF) [file pone.0017922.s005.tif]

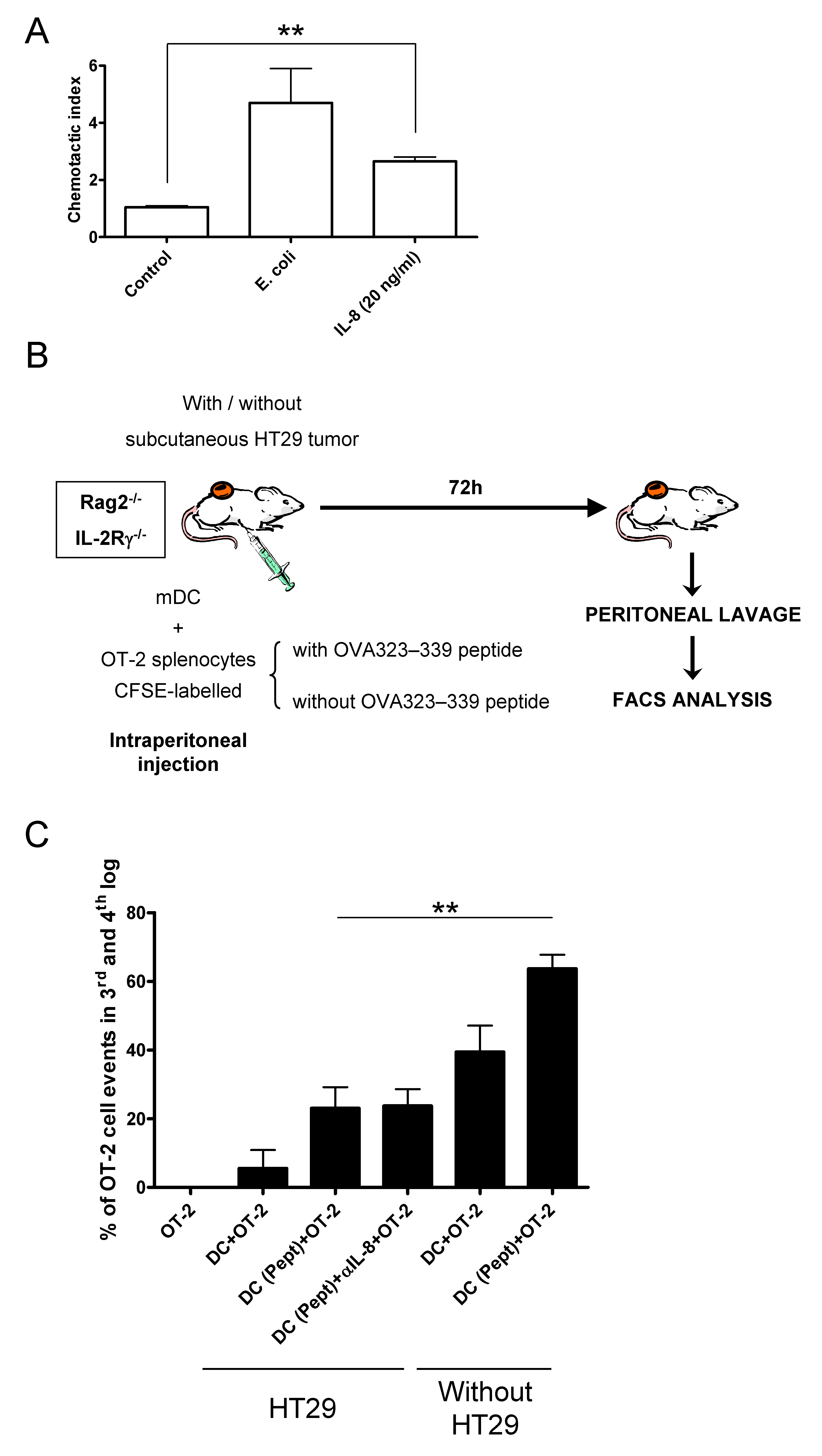

Supplement: Figure S6 — Activation of antigen specific murine CD4 T cells by DC in mice bearing HT29 tumors is suppressed by factors distinct from IL-8. (A) Mouse BM-derived DC [50], [55] were subjected to classical transwell chemotaxis assays towards culture medium (control), 105 heat-inactivated E. coli bacteria used as a positive control, or recombinant IL-8 as indicated. Data show a modest but reproducible attraction of mouse DC by human recombinant IL-8. (B) Schematic representation of experiments in which HT29-bearing Rag−/− IL-2Rγ−/− mice were injected in the peritoneal cavity with 5×10^6^ CFSE-labelled CD4 OT-2 cells [56] and 106 syngeneic DC pulsed with the OVA323-339 synthetic peptide. (C) Assessment of OT-2 T-cell proliferation by dilution of CFSE as in figure 4. Experiments were performed in mice bearing or not HT29 tumors with or without cognate peptide stimulation by the DC (n = 3 mice per group). When indicated 100 µg of anti-IL8 mAb were co-injected into the peritoneal cavity. (TIF) [file pone.0017922.s006.tif]

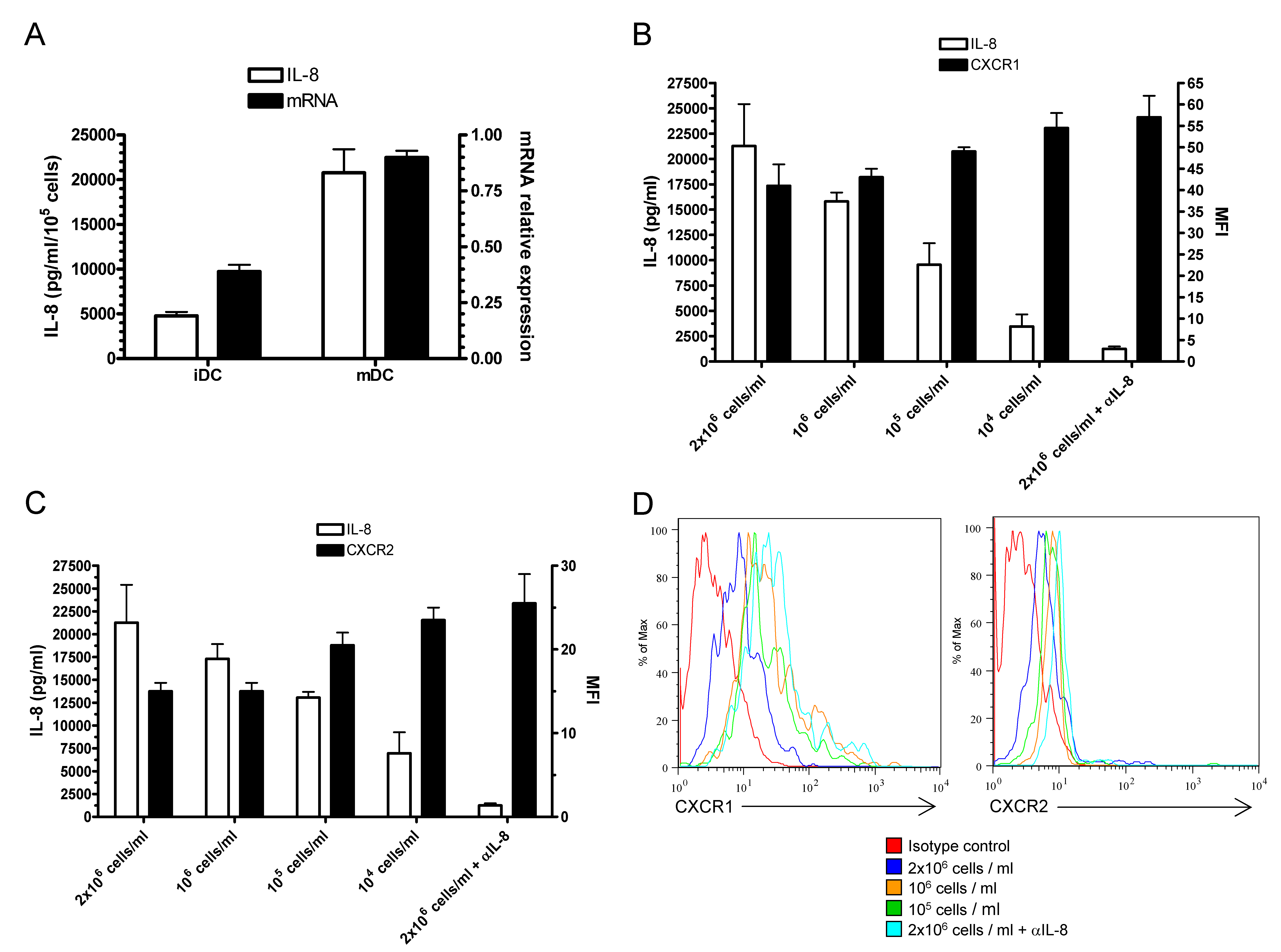

Supplement: Figure S7 — DC produce IL-8 and such autocrine IL-8 modulates in part the surface expression of CXCR1 and CXCR2. (A) Left axis: IL-8 concentration in the supernatant of mature (mDC) and immature DC (iDC); Right axis: mRNA encoding IL-8 in the corresponding DC cultures assessed by semi quantitative RT-PCR. (B and C) IL-8 concentration in the supernatant (left axes) and CXCR1 (B) and CXCR2 (C) surface expression as mean fluorescence intensity (MFI) analyzed by FACS (right axes). In B and C a mAb neutralising IL-8 (20 µg/ml) was added when indicated, or the DC were cultured under gentle agitation at the cellular densities given. Results are presented as mean±SD from triplicate experiments. IL-8 neutralisation or lower IL8 concentrations in the supernatants correlate with higher MFIs for CXCR1 and CXCR2 on the DC. (D) Shows representative FACS histograms from B and C. (TIF) [file pone.0017922.s007.tif]

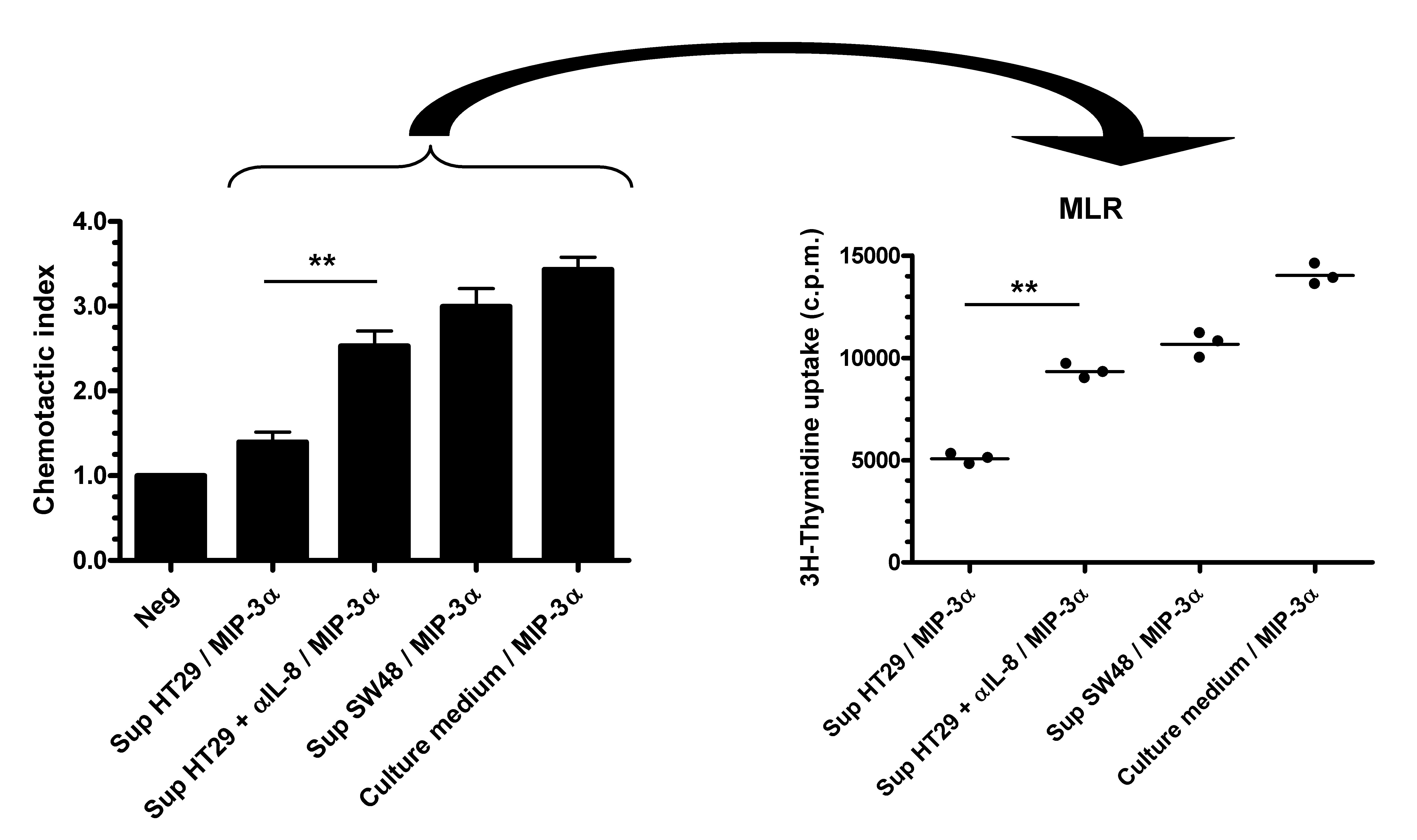

Supplement: Figure S8 — IL-8 in conditioned supernatants from HT29 cells impedes DC from migrating to MIP3α gradients. As a consequence fewer DC reaching the lower chamber results in less T-cell allostimulatory activity at this location. In order to model whether DC-disoriented migration would give rise to less T cell stimulation, we set up in the left panel chemotaxis assays in which DC migrated towards recombinant MIP3α (100 µg/ml). Data are presented as mean±SD of the chemotactic index normalized with culture medium without MIP3α (Neg). DC were seeded in the upper chamber with or without conditioned medium of HT29 cells or SW48 cells as indicated. When indicated an IL-8 neutralising antibody was added. In the right panel DC recovered from the lower chamber were used to stimulate allogenic PBL, and T-cell proliferation was recorded three days later as c.p.m. in 3H-Thy incorporation assays. Data represent three independent replicates. (TIF) [file pone.0017922.s008.tif]

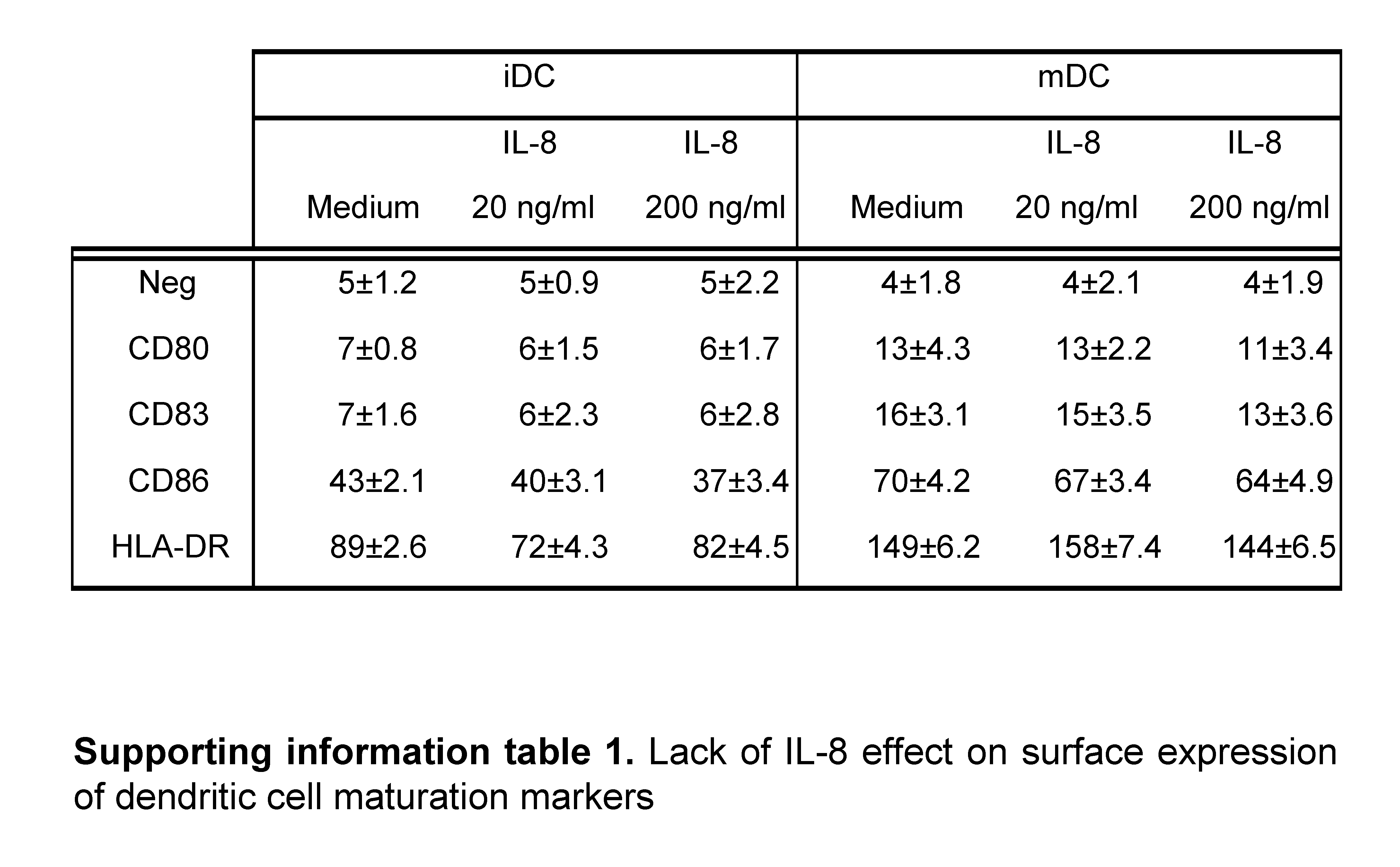

Supplement: Table S1 — Lack of IL-8 effect on surface expression of dendritic cell maturation markers. Mean fluorescence intensity of the indicated surface markers of DC upon FACS analyses in human DC (mean±SD from three different experiments) using either immature (iDC) or LPS+R848 matured DC (mDC), that were cultured in the absence or the presence of increasing concentrations of IL-8 as indicated in the columns. Experiments are representative of three similarly performed with different donors. (TIF) [file pone.0017922.s009.tif]
